# Traits linked to natural variation of sulfur content in *Arabidopsis thaliana*

**DOI:** 10.1093/jxb/erad401

**Published:** 2023-10-13

**Authors:** Nicholas de Jager, Varsa Shukla, Anna Koprivova, Martin Lyčka, Lorina Bilalli, Yanrong You, Jürgen Zeier, Stanislav Kopriva, Daniela Ristova

**Affiliations:** Institute for Plant Sciences, Cluster of Excellence on Plant Sciences (CEPLAS), University of Cologne, D-50674 Cologne, Germany; Institute for Plant Sciences, Cluster of Excellence on Plant Sciences (CEPLAS), University of Cologne, D-50674 Cologne, Germany; Institute for Plant Sciences, Cluster of Excellence on Plant Sciences (CEPLAS), University of Cologne, D-50674 Cologne, Germany; Mendel Centre for Plant Genomics and Proteomics, Central European Institute of Technology (CEITEC), Masaryk University, 625 00 Brno, Czech Republic; National Centre for Biomolecular Research, Faculty of Science, Masaryk University, 625 00 Brno, Czech Republic; Institute for Plant Sciences, Cluster of Excellence on Plant Sciences (CEPLAS), University of Cologne, D-50674 Cologne, Germany; Institute for Molecular Ecophysiology of Plants, Cluster of Excellence on Plant Sciences (CEPLAS), Heinrich Heine University, Universitätsstraße 1, D-40225 Düsseldorf, Germany; Institute for Molecular Ecophysiology of Plants, Cluster of Excellence on Plant Sciences (CEPLAS), Heinrich Heine University, Universitätsstraße 1, D-40225 Düsseldorf, Germany; Institute for Plant Sciences, Cluster of Excellence on Plant Sciences (CEPLAS), University of Cologne, D-50674 Cologne, Germany; Institute for Plant Sciences, Cluster of Excellence on Plant Sciences (CEPLAS), University of Cologne, D-50674 Cologne, Germany; Bielefeld University, Germany

**Keywords:** *Arabidopsis thaliana*, gene expression, glucosinolates, glutathione, natural variation, nutrients, sulfur

## Abstract

Sulfur (S) is an essential mineral nutrient for plant growth and development; it is important for primary and specialized plant metabolites that are crucial for biotic and abiotic interactions. Foliar S content varies up to 6-fold under a controlled environment, suggesting an adaptive value under certain natural environmental conditions. However, a major quantitative regulator of S content in *Arabidopsis thaliana* has not been identified yet, pointing to the existence of either additional genetic factors controlling sulfate/S content or of many minor quantitative regulators. Here, we use overlapping information of two separate ionomics studies to select groups of accessions with low, mid, and high foliar S content. We quantify series of metabolites, including anions (sulfate, phosphate, and nitrate), thiols (cysteine and glutathione), and seven glucosinolates, gene expression of 20 genes, sulfate uptake, and three biotic traits. Our results suggest that S content is tightly connected with sulfate uptake, the concentration of sulfate and phosphate anions, and glucosinolate and glutathione synthesis. Additionally, our results indicate that the growth of pathogenic bacteria is enhanced in the *A. thaliana* accessions containing higher S in their leaves, suggesting a complex regulation between S homeostasis, primary and secondary metabolism, and biotic pressures.

## Teaser

Biotic and abiotic environmental stressors trigger adaptations of sulfur homeostasis in *A. thaliana* leading to large variation in sulfur content in leaves of different accessions.

## Introduction

Sulfur (S) is an essential mineral nutrient for plant growth and health; however, research on S signaling and homeostasis is lacking compared with other macronutrients. This is mostly because S deficiency in modern agriculture was not an issue until recently (Bouranis *et al.*, 2020). Due to the implementation of the 1970 Clean Air Act and further 1990 Amendments, as well as a significant reduction of atmospheric S emissions (Likens *et al.*, 2001), S deficiency is becoming a threat to modern agricultural practice, especially when combined with other deficiencies (Jobe *et al.*, 2019). Apart from the importance of S for primary metabolism (Kopriva, 2015), S plays an important role in secondary metabolism and the synthesis of specialized plant metabolites that are crucial for biotic and abiotic interactions (Erb and Kliebenstein, 2020). Moreover, plant secondary metabolites can act as regulators of development, growth, and defense, and can be reallocated to primary metabolism (Erb and Kliebenstein, 2020).

Physiological traits in *Arabidopsis thaliana* natural accessions vary considerably (Koornneef *et al.*, 2004). This has evolutionary importance as some traits may be advantageous or provide adaptive value to a species under certain environmental conditions. Thus, there are multiple strategies by which plants can react to different stimuli to increase growth and yield. For example, in a study investigating the acclimation of different *A. thaliana* accessions to long-term nitrogen (N) limitation, it was found that vegetative shoot growth, root system architecture, and several other factors varied in response to low N (Ikram *et al.*, 2012; Meyer *et al.*, 2019). This suggests that multiple genetic factors are at play in response to different environmental conditions and that the evolutionary importance of these factors may fluctuate in different scenarios. Similar findings were also reported by North *et al.* (2009), who investigated nutrient use efficiency in low and normal N media. They showed that Arabidopsis accessions differed in levels of growth reduction in low N environments. It seemed that accessions with higher tolerance to N deficiency were capable of down-regulating nitrate reduction rates during periods of low N, thereby preserving nitrate reserves and maintaining fresh weight (North *et al.*, 2009). S content in leaves varies considerably in *A. thaliana*, with a range of up to 6-fold in plants grown under controlled conditions and optimal supply (Baxter *et al.*, 2007; Campos *et al.*, 2021). These variations may be signs of differing adaptive strategies being employed by plants facing different environmental stimuli. Unlike nutrients such as nitrate and phosphate, the mechanisms of regulation of sulfate homeostasis are still poorly understood and only a handful of investigations into how sulfate content is controlled have been conducted (Ristova and Kopriva, 2022). Loudet *et al*. (2007) found two major quantitative trait loci (QTLs) for sulfate content using Bay-0 and Shahdara recombinant inbred lines (RILs). The first QTL, identified as an APR2 isoform of adenosine 5ʹ-phosphosulfate reductase (APR), is a key enzyme involved in the sulfate assimilatory reduction pathway and it was found to be one of the determinants of the accumulation of sulfate (Loudet *et al.*, 2007). Loss of the activity of this enzyme, which was due to a substitution of Ala399 to Glu399, rendering the APR2 enzyme 99.8% inactive, caused the buildup of sulfate in the plant (Loudet *et al.*, 2007). Two additional weak alleles of *APR2* were independently associated with high sulfate/S content in Hod and Love-1 accessions, pointing to the importance of this gene for sulfate homeostasis (Chao *et al.*, 2014). A follow-up investigation by Koprivova *et al*. (2013), focusing on the second major QTL, identified an ATPS1 isoform of ATP sulfurylase (ATPS) as an additional regulator of sulfate levels in *A. thaliana*. ATPS is the preceding enzyme in the sulfate reduction pathway to APR, activating the sulfate by adenylation to adenosine 5ʹ-phosphosulfate (APS). The analysis revealed a difference in expression level among the RILs as well as Arabidopsis accessions, leading to variation in activity, and an increase of sulfate levels associated with low transcript accumulation (Koprivova *et al*., 2013). Although these findings were major steps forward in understanding the regulation of sulfate concentration in Arabidopsis, further investigation revealed that the QTLs containing these genes, SO10.1 (*APR2*) and SO10.2 (*ATPS1*), only explained 21% and 23% of the sulfate variation, respectively (Loudet *et al.*, 2007). Thus, additional genetic factors at work in controlling sulfate/S content in Arabidopsis remain undiscovered. One possible scenario is that a persistent presence of a stress (e.g. a herbivore or a pathogen) might result in an increased use of S from the environment, and increase the S content in those genotypes, in order to synthetize more specialized metabolites to repel the herbivore or pathogen. Another possible scenario is that because of the same stress, the genotype might adapt to use S more efficiently for synthesizing specialized metabolites, and therefore the total available content of S/SO_4_^2−^ in the leaves might be reduced.

Indeed, natural variation in S-containing secondary metabolites has been extensively studied in Arabidopsis (Kliebenstein *et al*., 2001; Sonderby *et al.*, 2007; Wentzell *et al.*, 2007; Chan *et al.*, 2010; Jimenez-Gomez *et al.*, 2011). The best example is the glucosinolates (GSLs), synthesized by the *Brassicaceae* family (including *A. thaliana*), which are the primary defence compounds against herbivores and pathogens (Burow and Halkier, 2017). Upon biotic attack, GSL levels can be induced, but this induction is dynamic and can lead to different pattern changes in GSLs, depending on the biotic and environmental factors (Del Carmen Martinez-Ballesta *et al.*, 2013; Yang *et al.*, 2020). *Arabidopsis thaliana* accessions exhibit extensive natural variation in GSLs due to multiple factors such as genetics, local environment, and population history (Katz *et al.*, 2021), suggesting that diversification of GSLs probably plays a vital role in local adaptation to biotic factors. GSL patterns are controlled by several major causal loci, including *AOP2*, *AOP3*, *MAM1*, *MAM3*, and *GSL-OH* (Chan *et al.*, 2010; Brachi *et al.*, 2015; Katz *et al.*, 2021). However, plant resistance to pathogens is polygenic, with numerous defence-related genes involved in the biosynthesis of defence compounds and cell wall modifications. This complexity of plant–pathogen interactions suggests that numerous genes underlie and contribute to the quantitative resistance to specific pathogens (Corwin *et al*., 2016; Corwin and Kliebenstein, 2017).

In this study, using a combination of quantitative genetics and systems biology tools, novel genetic regulators were identified for S homeostasis. Accessions with higher foliar S content showed higher rates of sulfate uptake, higher amount of GSLs, and higher susceptibility to pathogen attack. However, the same group of accessions showed lower foliar phosphate content and glutathione (GSH), and negatively correlated with latitude. Our results suggest that natural variation of S content is complexly interconnected with homeostasis of other nutrients, such as Pi, and primary and secondary metabolism (GSH and GSLs) driven by environmental biotic factors.

## Materials and methods

### Accession selection

In order to establish clusters of *A. thaliana* accessions with high versus low S content in their leaves, we re-analyzed ionomics data from Baxter *et al.* (2007) and Campos *et al.* (2021), with 174 accessions overlapping in the two datasets (Baxter *et al.*, 2007; Campos *et al.*, 2021). The two datasets of Baxter *et al.* (2007) and Campos *et al.* (2021) have different ranges of S concentrations, and individual accessions differ in absolute value but are usually in a similar percentile or relative range, with some exceptions. Therefore, we aimed to use only the overlapping accessions and to use both datasets to decide which accessions to choose. From these 174 overlapping accessions, a principal component analysis (PCA) was performed on the total of 37 ion data (Supplementary Table S1) to establish groupings of accessions with similar leaf S content. Based on the availability of these accessions, we selected 20 accessions with low, medium, and high sulfate levels for further investigation (Supplementary Fig. S1; Supplementary Table S1).

### Plant material and growth conditions

The seeds of the 16–18 Arabidopsis accessions used for sulfate uptake, gene expression analysis, and metabolite analysis were initially surface-sterilized with chlorine gas using 125 ml NaClO and 2.5 ml HCl (37%) for 3 h, after which sterile H_2_O was added for germination. The seeds were placed onto 0.8% agarose plates containing a modified Long-Ashton medium (Dietzen *et al.*, 2020). The medium consisted of 1.5 mM Ca(NO_3_)_2_·4H_2_O, 1 mM KNO_3_, 0.75 mM KH_2_PO_4_, 0.75 mM MgSO_4_·7H_2_O, and 0.1 mM Fe-EDTA in terms of macroelements. Microelements consisted of 10 µM MnCl_2_·4H_2_O, 50 µM H_3_BO_3_, 1.75 µM ZnCl_2_, 0.5 µM CuCl_2_, 0.8 µM Na_2_MoO_4_, 1 µM KI, and 0.1 µM CoCl_2_·6H_2_O. In the S-deficient medium, the 0.75 mM MgSO_4_·7H_2_O was replaced with a mixture of 0.7125 mM MgCl_2_·6H_2_O and 0.000225 mM MgSO_4_·7H_2_O, supplemented with 0.8 gl^–1^ MES salts, and 0.5% sucrose, and pH adjusted to 5.7 with KOH. The plates were placed at 4 °C for 2 d for optimum germination and then incubated vertically for 18 d in Sanyo light chambers with a photoperiod consisting of a 16 h:8 h light and dark cycle, with humidity at 60% and 21 °C. Alternatively, for determination of sulfate uptake, the plants were grown in 12-well plates as described in Koprivova *et al*. (2023). Sterile seeds were distributed onto square sterile nylon membranes and placed in 12-well plates on top of 1 ml of the modified Long-Ashton medium with 0.5% sucrose. After stratification for 2 d in the dark and cold, the plates were incubated for 3 d in the dark at 22 °C to promote etiolation, and further for 2 weeks in the growth cabinets under the long-day conditions as described above.

The bacterial pathogen *Burkholderia glumae* PG1 (Gao *et al*., 2015) was obtained from K.-E. Jäger, Heinrich Heine Universität Düsseldorf, Germany. The bacteria were kept as glycerol stock and plated freshly before the experiment on LB plates supplemented with chloramphenicol.

### Sulfate uptake

Sulfate uptake was measured in seedlings grown for 2 weeks in 12-well plates on a modified Long-Ashton medium under S-sufficient or S-deficient conditions. For uptake measurement, the medium was exchanged with 1 ml of the Long-Ashton medium with 0.2 mM sulfate supplemented with 12 mCi of [^35^S]sulfuric acid and incubated for 30 min in the light. Whole seedlings still on the mesh were washed thoroughly, blotted dry, and shoot and root samples were cut, weighed, and stored separately in liquid nitrogen until further processing on the same day. Samples were extracted in a 10-fold volume of 0.1 M HCl. A 10 μl aliquot of extract was used to determine sulfate uptake by scintillation counting (Dietzen *et al.*, 2020).

### RNA isolation and expression analysis

Total RNA was extracted from the roots of 18-day-old plants by standard phenol/chloroform extraction and LiCl precipitation. Thereafter, DNase treatment was performed and cDNA was synthesized from 600 ng of total RNA using the QuantiTect Reverse Transcription kit (QIAGEN) according to the manufacturer’s protocol. The product was diluted with autoclaved water to a final volume of 200 μl. Using quantitative real-time PCR (qPCR), 11 genes were examined using the gene-specific primers indicated in Supplementary Table S2. The qPCR was performed using the SYBR green as per the manufacturer’s instructions using a CFX96 Touch Real-Time PCR Detection System (Bio-Rad). All quantifications were normalized to the *TIP41* (AT4G34270) and *UBC21* (AT5G25760) genes using the 2^−ΔΔCt^ method. The RT-PCRs were performed in duplicate for each of the four independent samples.

### Metabolite analysis

#### Anions

For the measurement of phosphate, nitrate, and sulfate anions, 1 ml of sterile H_2_O was added to ~20 mg of homogenized shoot material, shaken for 1 h at 4 °C and heated to 95 °C for 15 min. The samples were centrifuged at maximum speed for 15 min at 4 °C, and 200 μl of the supernatants were transferred to an ion chromatography vial. Standard curves were generated using 0.5, 1, and 2 mM KH_2_PO_4_, KNO_3_, and K_2_SO_4_. The inorganic anions were measured with the Dionex ICS-1100 chromatography system and separated using a Dionex IonPac AS22 RFIC 43 250 mm analytic column (Thermo Scientific). The running buffer was made up of 4.5 mM NaCO_3_ and 1.4 mM NaHCO_3_ as described in Dietzen *et al.* (2020).

#### Thiols

To analyze low molecular weight thiols (Cys and GSH), ~20 mg of homogenized plant material was extracted with 0.1 M HCl at a 1:10 ratio (w/v) and subsequently centrifuged at maximum speed at 4 °C. To reduce the thiols in the samples, 60 μl of the supernatant was transferred to a new tube and 100 μl of 0.25 M CHES-NaOH (pH 9.4) was added. Thereafter, 35 μl of 10 mM DTT was added, and the tubes were vortexed and incubated for 40 min at room temperature. A 5 μl aliquot of 25 mM monobromobimane was added to the reduced extracts, and the samples were vortexed and incubated in darkness for 15 min at room temperature. The reaction was stopped by adding 110 μl of 100 mM methansulfonic acid and vortexing. After centrifugation at 4 °C for 20 min, 200 μl of supernatant was transferred into HPLC vials. Standards, ranging from 0 mM to 100 mM, were prepared using 2 mM l-Cys and GSH stocks. The conjugated thiols were resolved using reverse phase (RP)-HPLC (Eurospher 100-3 C18, 150 × 4 mm; Knauer) and a gradient of 90% (v/v) methanol and 0.25% (v/v) acetic acid, pH 4.1 in 10% (v/v) methanol and 0.25% (v/v) acetic acid, pH 4.1, and detected fluorimetrically with a 474 detector with an excitation wavelength at 380 nm and emission wavelength at 470 nm. The flow rate was constant at 1 ml min^−1^.

#### Glucosinolates

GSLs were extracted from ~20 mg of homogenized plant material using 500 ml of hot 70% (v/v) methanol, and 10 μl of sinigrin was added as internal standard. The extract was vortexed and incubated at 70 °C for 45 min, vortexing twice during the incubation. The samples were left to cool and centrifuged at maximum speed for 5 min at room temperature. The supernatant was transferred to prepared columns containing 0.5 ml of DEAE Sephadex A-25, washed twice with 0.5 ml of sterile H_2_O, and subsequently twice again with 0.5 ml of 0.02 M sodium acetate buffer. With a new tube placed underneath each column, a layer of 75 μl of sulfatase was placed onto the column. The samples were left at room temperature overnight, and the resulting desulfo-GSLs were eluted twice with 0.5 ml of sterile H_2_O, followed by a final elution by 0.25 ml. The eluates were combined, vortexed, centrifuged for 5 min, and 200 μl of the supernatant were transferred to HPLC vials. The desulfo-GSLs were resolved by HPLC (Spherisorb ODS2, 250 3 4.6 mm, 5 μm; Waters) using a gradient of acetonitrile in water and detected by UV absorption at 229 nm. The GSLs were quantified using the internal standard and response factors as described in Dietzen *et al.* (2020).

#### Camalexin

Camalexin was extracted from ~50 mg of leaves in 200 µl of dimethlysulfoxide (DMSO) as described in Koprivova *et al*. (2019). After centrifugation at room temperature for 20 min, 20 µl aliquots of the supernatant were injected into a Thermo Scientific Dionex UltiMate 3000 HPLC system with a Waters Spherisorb ODS-2 column (250 mm×4.6 mm, 5 µm). The samples were resolved using a gradient of acetonitrile in 0.01% (v/v) formic acid. Camalexin was detected by a fluorescence detector with an excitation at 318 nm and emission at 368 nm. For the quantification, external standards were used ranging from 1 pg µl^–1^ to 1 ng µl^–1^ (Koprivova *et al*., 2019).

### Assessment of plant resistance to *Pseudomonas syringae*

To assess bacterial growth in naive 5-week-old plants, overnight log phase cultures of *Pseudomonas syringae* pathovar *maculicola* were washed three times with 10 mM MgCl_2_ and diluted to a final optical density at 600nm (OD_600_)=0.001 before infiltrating the resulting bacterial suspensions from the abaxial side into three fully grown leaves using a 1 ml syringe without a needle. The infiltration was performed between 10.00 h and 11.00 h, as previously described (DOI: 10.1016/j.cell.2018.02.049). Approximately 60 h later, the bacterial growth was quantified by measuring the bacterial bioluminescence in leaf discs (10 mm in diameter) of infiltrated leaves (one disc per leaf, three discs per plant) using a Sirius FB12 luminometer (Berthold Detection Systems). For each independent experiment, at least 18 replicate leaves from 6–7 plants per genotype were measured before performing a statistical analysis of the resulting values.

### Co-cultivation with *Burkholderia glumae*

Plants were grown in the 12-well plates as described above for 14 d. For inoculation, overnight bacterial cultures were washed twice with sterile 10 mM MgCl_2_ and the final OD_600_ was measured. The *B. glumae* PG1 was diluted stepwise to OD_600_=0.0005 in 10 mM MgCl_2_. An 8 µl aliquot of these suspensions was used for inoculation into each well and 8 µl of 10 mM MgCl_2_ was used as mock treatment. Samples for camalexin (shoots) and DNA (roots) were harvested after 3 d of inoculation.

### Determination of bacterial titer

Bacterial titer in the roots was determined using the qPCR method as described in Koprivova *et al*. (2023). Genomic DNA was extracted by a buffer containing 0.025 M EDTA, 0.2 M Tris pH 8.0, 0.25 M NaCl, and 0.5% SDS. After 10 min incubation at 65 °C and centrifugation, the supernatant was precipitated with isopropanol, washed with 70% ethanol, and resuspended in 100 µl of sterile water. For the qPCR, 13 ng of corresponding DNA samples were used with Arabidopsis- (At primer AT4G26410) and *B. glumae* PG1-specific primer (Burk1 for NR042931). The qPCR conditions were the same as for expression analysis. The qPCRs were performed in duplicate for each of the four independent samples. The qPCR results were related to the bacterial titer as established in Koprivova *et al*. (2023).

### Statistical analysis

Initial raw data from the qPCR and HPLC experiments were processed using Excel software (Microsoft Office 365). These processed data were used for statistical analysis. All experiments made use of 3–4 biological replicates for each accession. The accessions were grouped into low, medium, and high sulfate groups, which were then used in a one-way ANOVA. Furthermore, a Tukey honest significant difference (HSD) post-hoc between significantly different groups was performed. The level of significance was set at *P*≤0.05.

For clustering analysis, we extracted ~400 genes, annotated in S, N, and phosphorus (P) homeostasis and metabolism (www.arabidopsis.org) from a previously published dataset (Kawakatsu *et al.*, 2016), and transformed the counts into z-scores on a gene-by-gene basis in the seven accessions, three from our low S content group, three from our high S content group, and the reference accession, Col-0. Multiple Experiment Viewer software (TIGR; http://mev.tm4.org) was used to create heat maps and perform cluster analysis using QTC with Pearson correlation, hierarchical clustering: average linkage method, and diameter 0.1. The pair-wise correlation analysis between the traits was performed using the ‘Hmisc’ and ‘corrplot’ packages in R (https://www.Rproject.org). A multivariate network was created in Cytoscape (Su *et al.*, 2014) based on significant pair-wise correlations between all quantified traits.

## Results

### Principal component analysis of sulfur content in leaves

**Fig. 1.**
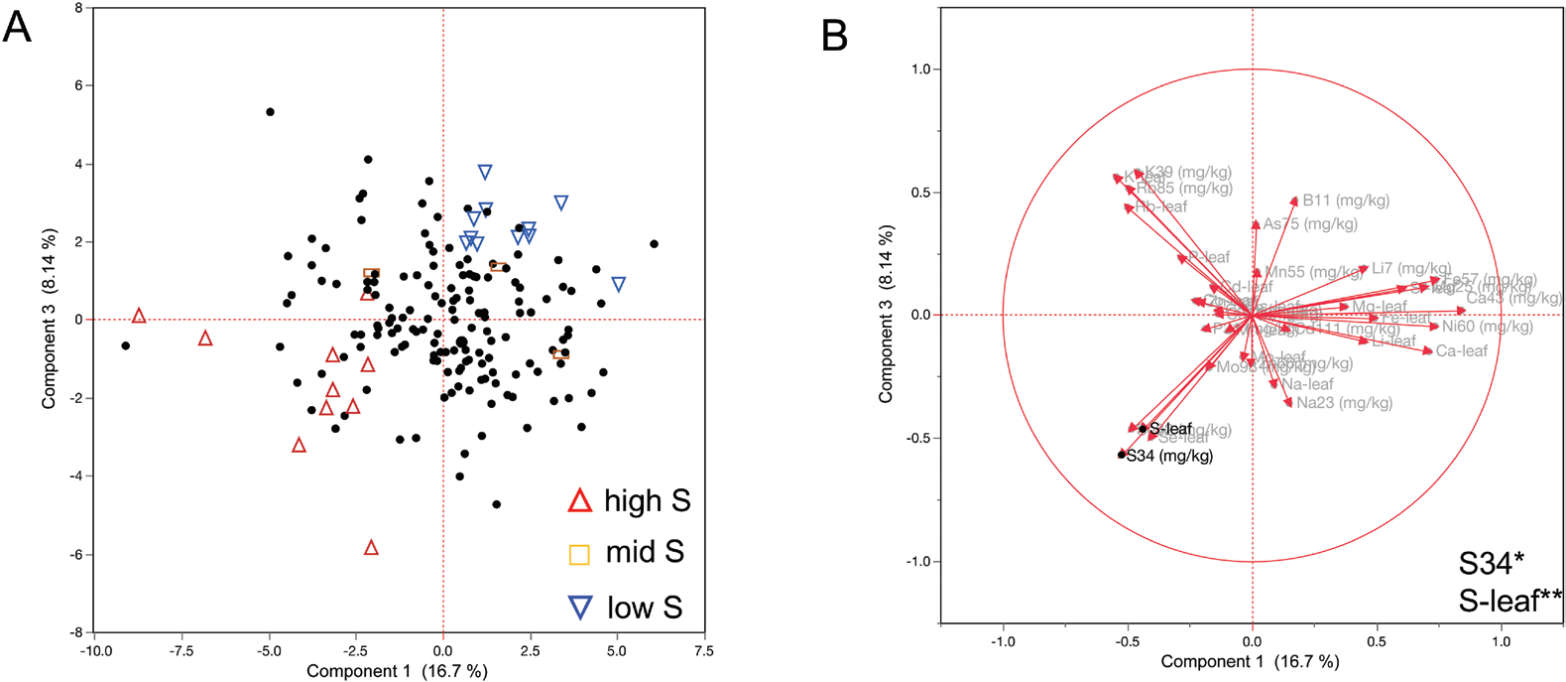
Natural variation of sulfur (S) content in *Arabidopsis thaliana* leaves in 174 accessions. (A) Principal component analysis on ionomics of 174 overlapping accessions between the two datasets (Baxter *et al.*, 2007; Campos *et al.*, 2021) Accessions that were chosen based on the PCA are marked with a red upright triangle for accessions with a high S content (Com-1, Rak-2, Uod-7, Zu-1, Pa-1, Di-1, Si-0, Tamm-2, Rou-0, Hod), a blue inverted triangle for accessions with a low S content (App1-16, Rhen-1, TDr-18, Jl-3, RRS-7, Gr-5, In-0, Lis-1, Bro1-6, T510, and T1110), and orange squares for accessions with a mid S content (Col-0, Kro-0, and Kulturen-1). (B) Ionomics trait loadings between PC 1 and PC 3 (*Baxter *et al.*, 2007; **Campos *et al.*, 2021). All values and accession information are listed in Supplementary Table S1. The principal components loading matrix and eigenvalues are listed in Supplementary Table S3.

Two ionomics datasets were reported with 350 and 1135 *A. thaliana* accessions, respectively, showing a large variation in elemental composition of the leaves, including S (Baxter *et al.*, 2007; Campos *et al.*, 2021). Using these datasets in genome-wide association studies (GWAS), numerous genes controlling variation in the concentration of certain elements have been identified (Baxter *et al.*, 2007; Campos *et al.*, 2021); however, this was not the case for S. Overlapping accessions among the two datasets showed significant variation in S content within the genotypes. Therefore, we used the 174 overlapping accessions, and re-analyzed them using the complete ionomics values from both studies (Supplementary Table S1). We performed PCA to identify the primary contributions (loadings) of the different elements, specifically S content. Our results indicated that the first three principal components (PCs) explain 33% of the variation, and S content has the highest contribution to the first and third PCs, compared with the other PCs (Fig. 1; Supplementary Table S3). Plotting the first and the third PCs against each other grouped the accessions according to low, medium, and high S content in their leaves (Fig. 1). Therefore, for further analysis to determine factors underlying the variation in foliar S, we used accessions from these groupings.

### Anion quantification in extreme accessions

**Fig. 2.**
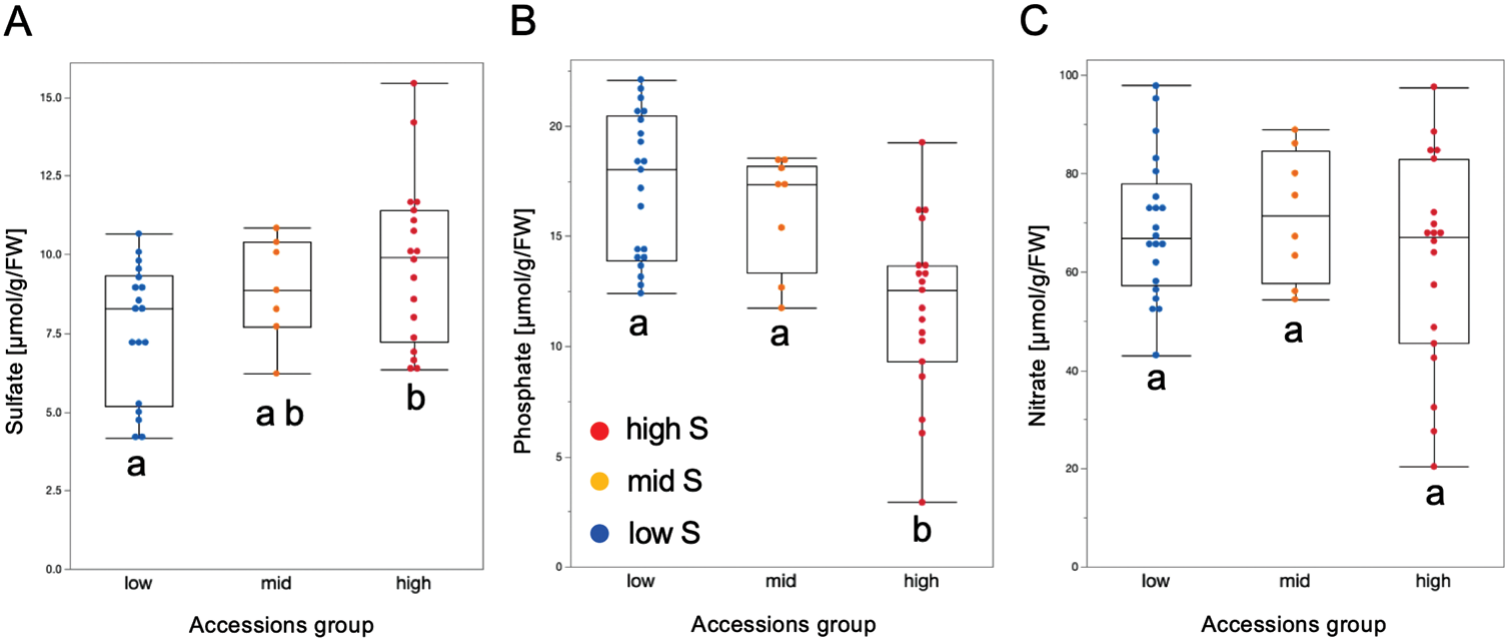
Quantification of anion content in leaves of three groups of *Arabidopsis thaliana* accessions. (A) Quantification of sulfate (SO_4_) anions (µmol g^–1^ FW). (B) Quantification of phosphate (PO_4_) anions (µmol g^–1^ FW). (C) Quantification of nitrate (NO_3_) anions (µmol g^–1^ FW). Plants were grown on vertical plates for 18 d, and shoots and roots were harvested separately. Shoots were used for anion quantification. Ten plants were plated on each plate, with four biological replicates per genotype. When harvesting, 10 shoots or 10 roots from each plate were bulked in one sample. Means ±SE are shown; significant differences between the groups are marked with different letters (*P*<0.05, ANOVA).

Accessions identified above with low, medium, and high S content were used to quantify the abundance of sulfate anions in leaves, in order to assess whether sulfate content correlates with S content, quantified previously. We found that, indeed, sulfate was significantly more abundant in the group of accessions with high S content (Fig. 2A; Supplementary Fig. S2). In addition, we measured phosphate and nitrate anions in the leaves. Interestingly, the opposite trend was found to be significant for phosphate, where the group with high S content had a lower amount of phosphate (Fig. 2B; Supplementary Fig. S2). For nitrate, we did not observe any differences among the groups. These findings suggest that S content positively associates with sulfate content in Arabidopsis accessions, while phosphate anions negatively correlate with total S content but not sulfate.

### Sulfate uptake is associated with sulfur content in leaves

**Fig. 3.**
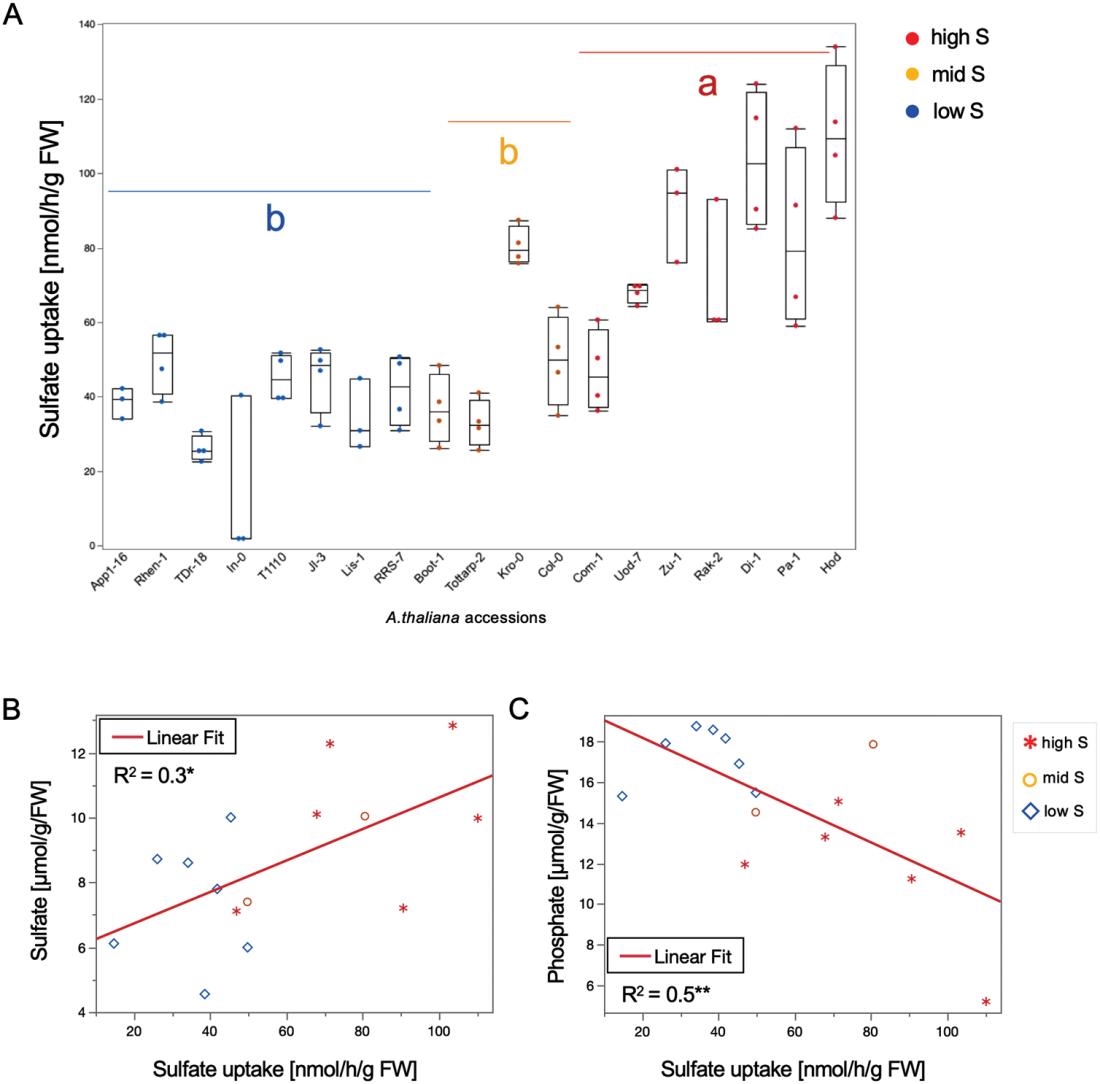
Sulfate uptake in *Arabidopsis thaliana* accessions. (A) Sulfate uptake in roots (nmol h^–1^ g^–1^ FW). (B) Regression analysis of sulfate anion content and sulfate uptake in contrasting accessions, based on leaf S content. Means ±SE (*n*=4) are shown; significant differences between the groups are marked with different letters (*P<0.05) ANOVA, Tukey test. (C) Regression analysis of phosphate anion content and sulfate uptake in contrasting accessions, based on leaf S content. Arabidopsis plants (25 seedlings) were grown on a nylon net in hydroculture for 14 d and, after incubation with [^35^S] sulfate for 30 min, shoots and roots were harvested separately and extracted with 0.1 M HCl for radioactivity quantification.

Plants acquire S mostly as sulfate through root absorption. Therefore, 18 accessions from the three groups of low, middle, and high S content in leaves were used to determine the rate of sulfate uptake. We observed significant differences in sulfate uptake among the three groups (Fig. 3A), as well as in the root-to-shoot translocation rate and the allocation of the sulfate taken up in shoots (Supplementary Fig. S3). The accession group with higher S content in leaves also had higher sulfate uptake (Fig. 3A). There was only a weak positive correlation (*R*^2^=0.3) between the uptake and sulfate content in the leaves (Fig. 3B). Interestingly, we found a stronger negative correlation of sulfate uptake with phosphate content (Fig. 3C). We also observed significant correlation of sulfate uptake with original S content in the two datasets, PCs 1 and 3, but not with PC 2, and nitrate anions (Supplementary Fig. S4). Thus, under normal growth conditions, it seems that the differences in sulfate and S content are at least partially driven by differences in sulfate uptake rate.

### Correlation of gene expression with sulfur content and sulfate uptake

**Fig. 4.**
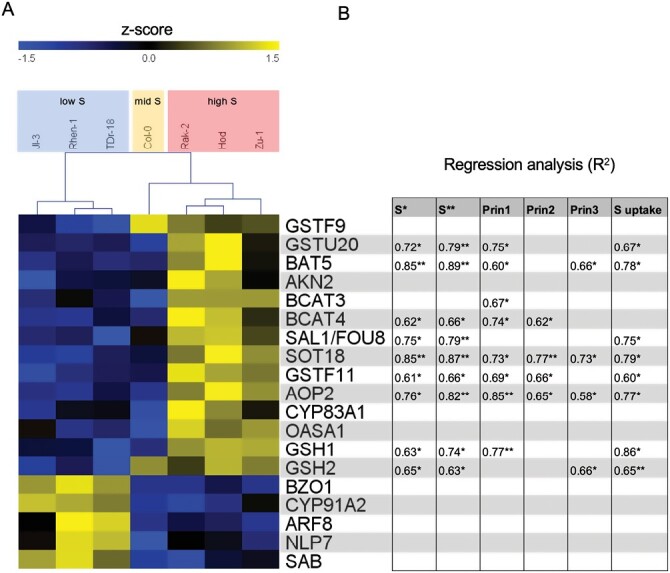
Expression profiles of contrasting *Arabidopsis thaliana* accessions, based on S content in leaves. (A) Expression profiles of genes related to nutrient homeostasis in seven contrasting accessions with low, mid, and high leaf S content (expression values were transformed into z-scores from Kawakatsu *et al.*, 2016). (B) Linear regression analysis was fitted between expression and S content (S*, Baxter *et al.*, 2007; S**, Campos *et al.*, 2021), the first three PCs (Supplementary Table S3), and S uptake (Fig. 3A). Genes that had significant *R*^2^ with at least three traits were considered for further qPCR validation.

To test whether the phenotypic differences observed among the contrasting accessions are reflected at the gene expression level, we made use of a public dataset of transcriptome data (Kawakatsu *et al.*, 2016) in seven accessions corresponding to the groupings of low, middle, and high S content from our PCA. We extracted ~400 genes implicated in nutrient (N, P, and S) signaling, transport, and homeostasis regulation, transformed the expression values to z-scores, and performed clustering analysis. Of interest were clusters that have opposite transcription output in the low S and high S accessions (Supplementary Table S4). We identified four clusters, of which three showed specific up-regulation of genes in the high S content group of accessions (Rak-2, Hod, and Zu-1), and one showed specific up-regulation of genes in the low S content group (Rhen-1, Jl-3, and TDr-18) (Supplementary Fig. S5). We then selected genes that have opposite patterns of expression in the seven accessions, and performed regression analysis using both datasets for S content (Baxter *et al.*, 2007; Campos *et al.*, 2021), three PCs, and S uptake (Fig. 4). Nine genes had asignificant *R*^2^ for at least three traits, all involved in GSL and GSH metabolism.

To extend the analysis to more genotypes, we performed qPCR on 14 accessions of all three groups. Beside the nine genes identified above, another 11 genes that were previously described to be regulated by sulfate supply and/or accumulation were analyzed (Loudet *et al.*, 2007; Koprivova *et al*., 2013); genes for sulfate transport and assimilation, and for phosphate transporters, because of the negative association of phosphate and sulfate uptake (Fig. 3C). For the sulfate transporter gene *SULTR1;1*, significant differences were observed between the high sulfate accessions and mid sulfate accessions, as well as between high sulfate accessions and low sulfate accessions, while *SULTR1;2* showed no significant differences between either group (Supplementary Fig. S6).

The initial phase of the sulfate assimilatory reduction pathway is controlled largely by the *ATPS1*, *APR2*, and *SiR* genes (Koprivova and Kopriva, 2014). Two of these genes, *APR2* and *ATPS1*, that were previously reported to underlie natural variation of sulfate content in Arabidopsis (Loudet *et al.*, 2007; Koprivova *et al*., 2013), showed some increase in their relative expression in the roots of higher S accessions, but not significant at the chosen level (Supplementary Fig. S6). Similarly, *SiR*, the gene encoding sulfite reductase responsible for sulfite reduction to sulfide, one of the later steps in the pathway (Koprivova and Kopriva, 2014), showed no significant difference between the groups.

Sulfotransferases play a key role in biosynthesis of sulfated secondary metabolites, such as GSLs. In our *in silico* analysis, only *SOT18* showed significant association of expression with all traits (Fig. 4), but we also tested *SOT17* and *SOT16* because of their redundant function (Piotrowski *et al.*, 2004). Both *SOT17* and *SOT18* genes were found to be expressed significantly more highly in the high S accessions compared with the low S accessions, especially *SOT18*, but this was not true for *SOT16* (Supplementary Fig. S6). Other genes that belong to biosynthetic pathway of GSLs showed significant differences between the low and high groups of accessions, namely *BCAT4* and *BAT5* that are involved in the first and second step of the biosynthesis of methionine-derived GSLs (Supplementary Fig. S6) (Schuster *et al.*, 2006; Sawada *et al.*, 2009).

Several genes involved in GSH metabolism showed significant association of expression with multiple traits among the seven accessions (Fig. 4). However, in our qPCR analysis with 14 accessions from the low, mid, and high S content, only *GSTF11* encoding GSH-S-transferase showed significant differences between the accession groups (Supplementary Fig. S6).

Two phosphate-related genes, *PHT1;1*, encoding a high-affinity phosphate transporter that is involved in phosphate uptake from soil as well as translocation from roots to leaves (Ayadi *et al.*, 2015) and underlies natural variation of phosphate uptake (Chien *et al.*, 2022); and *PHO2* (*PHOSPHATE 2*), a ubiquitin-conjugating E2 enzyme involved in phosphate starvation response and mediating degradation of PHO1 and PHT1s (Liu *et al.*, 2012), showed significant differences between the groups (Supplementary Fig. S6).

In summary, the gene expression analysis showed that multiple genes associated with sulfate uptake and S metabolism, including sulfate transporter (*SULTR1;1*), genes involved in GSL synthesis (*BCAT4*, *BAT5*, *SOT17*, and *SOT18*), GSH metabolism (*GSTF11*), and phosphate transport and signaling (*PHT1;1* and *PHO2*), showed significant differences among the 14 accessions contrasting in S accumulation.

### Variation in sulfur-containing metabolites

Important S-containing metabolites, such as the thiols Cys and GSH, were also quantified in the contrasting accessions. Cys is considered a keystone compound connecting S, N, and carbon metabolism (Jobe *et al.*, 2019). Our findings suggest that the ability to take up sulfate in different accessions does not affect Cys accumulation in Arabidopsis, as Cys levels varied between high, low, and mid S accessions, and no significant difference was detected among the groups (Supplementary Fig. S7A). Similarly, to phosphate anions, GSH showed a significantly lower content in high S-containing accessions in comparison with low S accessions (Supplementary Fig. S7B).

The biosynthesis of GSLs is complex and involves many secondary modifications that result in a multitude of differing GSLs (Blazevic *et al.*, 2020). Also, GSL profiles vary in Arabidopsis dependent on several QTLs (Kliebenstein *et al*., 2001; Sonderby *et al.*, 2007; Wentzell *et al.*, 2007; Chan *et al.*, 2010; Jimenez-Gomez *et al.*, 2011). We revealed a significant difference between the grouped accessions, specifically between the high and low S groups, where high S accessions contained more GSLs (Supplementary Fig. S8). Detailed analysis revealed that this difference was significant for both aliphatic and indolic GSLs, with the highest differences for I3M (indol-3-ylmethyl) and 4-MOIM (4-methoxyindol-3-ylmethyl) that belong to the indolic class of GSLs (Supplementary Fig. S8; Supplementary Table S5).

Since we observed significant differences in GSLs between the low and high S groups of accessions, as well as for most of the individually quantified GSLs and GSH, we were interested in which metabolites are significantly associated with sulfate uptake. Thus, we performed a linear regression analysis, and found that sulfate uptake is significantly positively associated with indolic GSLs, specifically I3M, and negatively associated with GSH content (Supplementary Fig. S9). Interestingly, when calculating the total amount of S atoms in each GSL, we found that only I3M has a significant positive correlation with total S (Baxter *et al.*, 2007), sulfate uptake, and sulfate anions (Supplementary Fig. S10).

### Multivariate analysis of sulfur content, sulfate uptake, gene expression, and metabolites

**Fig. 5.**
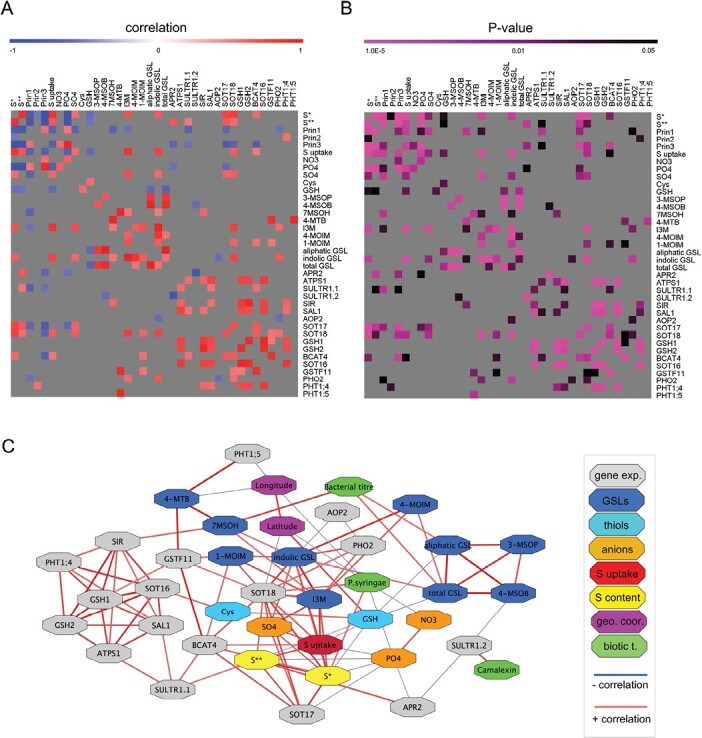
Correlation analysis and network analysis of multiple variables. (A) Correlation matrix of sulfur-related trait data in three contrasting accession groups with low, mid, and high S content in leaves. The results are presented as a heat map with the Pearson correlation score from –1 to 1 and (B) significant *P*-values (*P*<0.05). The correlation coefficients and *P*-values are given in Supplementary Tables S6 and S7. (C) A multivariate network was created based on significant pairwise correlations between all quantified variables. All significant pairwise correlations are listed in Supplementary Table S8. S* content, Baxter *et al.* (2007); S** content, Campos *et al.* (2021). Principal components 1–3 (Prin1–3): Supplementary Table S1. Tyols: cysteine (Cys), gluthatione (GSH). Anions: sulfate (SO_4_^2−^), phosphate (PO_4_^2−^), nitrate (NO_3_^−^). Glucosinolates (GSLs): 3-MSOP (3-methylsulfinylpropyl), 4-MSOB (4-methylsulfinylbutyl), 7MSOH (7-methylsulfinylheptyl), 4-MTB (4-methylthiobutyl), I3M (indol-3-ylmethyl), 4-MOIM (4-methoxyindol-3-ylmethyl), 1-MOIM (1-methoxyindol-3-ylmethyl). Biotic traits: camalexin, bacterial titer, and plant resistance to *P. syringae*.

To identify significant association between the multiple traits among the three groups of accessions (low, mid, and high S content), we performed multiple correlation analysis, and identified >100 significant pairwise correlations (Fig. 5A; Supplementary Tables S6, S7). Sulfate uptake showed significant correlation with both ionomics datasets (Baxter *et al.*, 2007; Campos *et al.*, 2021), first and third PCs (Fig. 5A, B), phosphate content, and expression of two genes (*BCAT4* and *SOT18*) both involved in GSL synthesis (Mitreiter and Gigolashvili, 2021). Sulfate uptake was also positively correlated with accumulation of I3M, but negatively with GSH and phosphate anions (Fig. 5; Supplementary Tables S6, S7).

The transcript level of *SOT18* showed significant positive correlations with sulfate uptake and S content in the accessions (Fig. 5A, B; Supplementary Fig. S8). Additionally, *SOT18* positively correlated with the most abundant indolic GSL, I3M; whereas *SOT16* and *SOT17* did not. *SOT17* is known to be less specific to I3M than *SOT18* (Klein and Papenbrock, 2009). Interestingly, *SOT17* also correlated negatively with the phosphate levels, while *SOT18* correlated positively with *PHO2*, indicating a possible connection between the P and secondary S metabolism.

The phosphate levels were also negatively correlated with the S content (both datasets), sulfate uptake, and *SOT17* (Fig. 5). These findings could indicate an antagonistic effect between phosphate and sulfate homeostasis and metabolism, or a need for plants to maintain an anionic balance. The uptake and metabolism of nitrate may also be involved as these were positively correlated with phosphate levels (Fig. 5; Supplementary Table S7).

### Probing the immunity response in accessions differing in sulfur content

Since we found that S content and sulfate uptake correlate with indolic GSLs, and expression of several genes involved in GSLs biosynthesis, which function as primary defense compounds against herbivores and pathogens (Burow and Halkier, 2017), we next intended to find out if accessions with different foliar contents of S also show different responses to pathogens. First, we tested the plant resistance to infection with *Pseudomonas syringae*, as previously described (Hartmann *et al.*, 2018). Although we observed a trend of increased susceptibility in the accessions with higher S content, and the most susceptible accession was Hod with the highest S content (Supplementary Fig. S11), these differences were not significant, since we observed high variation within the same accessions. As an alternative, we tested whether camalexin synthesis and bacterial titer after root infection with a bacterial pathogen were affected by S status; to this end, we grew the accessions in hydroculture with *B. glumae* PG1 (Koprivova *et al*., 2023). We found that camalexin production in the shoot was not significantly different among the three groups of accessions (Supplementary Fig. S12A). However, the bacterial titer in the roots was significantly higher in the group of accession that had higher leaf S content (Supplementary Fig. S12B).

## Discussion

The S content in *A. thaliana* leaves varies up to 6-fold (Baxter *et al.*, 2007; Campos *et al.*, 2021), and these variations indicate differing adaptive strategies to cope with different environmental stimuli. Several genes contributing to the control of this variation have been identified (Loudet *et al.*, 2007; Koprivova *et al*., 2013; Chao *et al.*, 2014), but they explain only ~20% of the variation. In addition, it is unclear what kind of environmental stimuli is driving this variation in S content. Here, we present evidence that the S content in leaves is tightly connected with sulfate uptake, the concentration of sulfate and phosphate anions, and GSL and GSH synthesis. Additionally, our results suggest that the growth of pathogenic bacteria is enhanced in the *A. thaliana* accessions containing higher S in their leaves, indicating that the ‘tug-of-war’ for nutrients also applies to S (Wang *et al.*, 2022).

### Sulfur content and sulfur-containing metabolites

S in plants is found in many forms, inorganic and organic, and it is not clear whether the variation in S content is reflected in the same way in the variation of these different S pools. The greatest pool of S, particularly in the *Brassicaceae*, is sulfate (Blake-Kalff *et al.*, 1998); therefore, the simplest hypothesis to explain the variation in total S would be a variation in sulfate content, particularly as the two genes identified to underlie the variation in S, *APR2* and *ATPS1*, do so through affecting primarily sulfate levels. However, while the accessions of the high S group have higher sulfate content than those of the low S group, there is only a weak correlation between sulfate and S content of one of the ionomics experiments (Fig. 5) (Baxter *et al.*, 2007). On the other hand, sulfate uptake correlates with S content from both ionomics datasets, but, surprisingly, not with sulfate content (Fig. 5). Arabidopsis accessions thus must vary in the sulfate utilization efficiency (i.e. in the ratio between organic and inorganic S) and, therefore, in the allocation of taken up sulfate between storage and metabolism. Understanding the control of this trait would be particularly important for crop plants, to prevent excessive storage of sulfate and reduce the use of S fertilizer.

Interestingly, when searching for an opposite pattern of expression in the low, mid, and high S groups of accessions, we found that genes involved in GSL and GSH metabolism were over-represented (Fig. 4), suggesting that leaf S content is tightly interconnected with primary and secondary S metabolites. Indeed, many secondary metabolites decrease during nutrient limitations. For instance, GSL biosynthesis is reduced under low S conditions, probably due to the prioritization of S for primary S metabolism (Aarabi *et al.*, 2016; Maruyama-Nakashita, 2017), suggesting that retrograde flow from secondary metabolism back to primary metabolism is crucial for the plant. In fact, recently it was demonstrated that S atoms from GSLs are reallocated back to primary metabolites, such as Cys in *A. thaliana* (Sugiyama *et al.*, 2021). Therefore, more evidence is supporting the scenario where the secondary metabolites are not just a peripheral appendage, but are an integral part of the plant metabolism (Angelovici and Kliebenstein, 2022). In addition, natural variation in GSL accumulation in Arabidopsis has long been established, as well as a few genetic loci controlling this variation (Chan *et al.*, 2010; Brachi *et al.*, 2015; Katz *et al.*, 2021). However, none of these QTLs is directly connected to S, even though S homeostasis clearly affects GSL contents. The limitation in synthesis of activated sulfate, PAPS, in APS kinase mutants resulted in strongly reduced GSL levels (Mugford *et al*., 2009). Also, the *sultr1;2* mutant (*sel1-10* allele), which is deficient in sulfate uptake, accumulates lower levels of GSLs in the leaves (Morikawa-Ichinose *et al*., 2019). Thus, it could be expected that accumulation of GSLs might be also linked to S status of the accession, but on the quantitative rather than the qualitative level, and more probably for the aliphatic GSLs that require more S for their synthesis. This has, however, not been the case, and the only GSL that correlated with S uptake was the indolic I3M (Fig. 5). On the other hand, the fact that the variation in GSL profiles is not reflected in the variation in S can be explained by the relatively small portion of S found in the GSLs compared with other S pools.

Glutathione (GSH; l-γ-glutamyl-l-cysteinyl-glycine) is an important storage of Cys, and the main transport form of reduced S (Kuzuhara *et al.*, 2000; Leustek *et al.*, 2000). GSH is part of the redox system preventing cell damage, by reversibly undergoing redox reactions at its thiol group, and thus maintaining the redox status of the cell (Noctor *et al.*, 2012). Similarly to GSLs, under S-limiting conditions, GSH is decreased by 3-fold in *A. thaliana* shoots (Dietzen *et al.*, 2020), suggesting that S-deficient plants are more vulnerable to both abiotic and biotic stress factors. However, GSH and GSLs are tightly connected, as GSH provides reduced S for GSL synthesis, and can indirectly affect GSL synthesis (Geu-Flores *et al.*, 2009). Our results suggest that GSH and GSLs are regulated in an opposite way; namely, accessions with higher S and sulfate content, higher GSL content, and higher sulfate uptake had lower GSH content and also lower phosphate anions (Figs 2, 3; Supplementary Figs S7, S8). Although phosphate deprivation does not affect total GSH content in plants, it increases the reduced to oxidized ratio (GSH/GSSG) (Trujillo-Hernandez *et al.*, 2020). The *cad2* mutant, affecting the GSH1 rate-limiting enzyme in GSH synthesis, showed hyposensitivity to phosphate deprivation, but the clear connection between phosphate homeostasis and GSH synthesis and/or redox state remains unclear (Trujillo-Hernandez *et al.*, 2020). In some species such as crayfish, GSH activity correlated with species origin latitude (Fanjul-Moles *et al.*, 2003), suggesting that local environmental abiotic and biotic factors are driving this variation. GSH levels are known to follow diurnal and seasonal cycles in some plant species, and high levels of GSH in the winter are linked to winter hardiness and protection against winter injury (Turunen and Latola, 2005). We have observed an ~3-fold variation in both Cys and GSH contents in the small set of accessions analyzed (Supplementary Fig. S7), pointing to a substantial variation in these metabolites across the populations. Whether on a larger scale this variation would correlate with S and what are the genetic loci driving this variation need to be determined.

### Significance of sulfur metabolism for plant–bacteria interactions

The role of S in plant–pathogen interactions became evident at the beginning of the 19th century when S was recommended as an effective fungicide (Bloem *et al.*, 2005). However, the role of S in the resistance of crops against various pathogens became clear during the 1980s, when the Clean Air legislation was mandated and the atmospheric S depositions were significantly reduced (Likens *et al.*, 2001), leading to increased susceptibility to diseases, particularly for high S-demanding crops (Bloem *et al.*, 2014).

Sulfate uptake and metabolism are generally important for plant interaction with bacteria. In the case of nitrogen-fixing rhizobia, sulfate transport from the plant host across the symbiosome membrane is high, and the *Lotus japonicus* sulfate transporter SST1 has an important role in this symbiotic interaction (Krusell *et al.*, 2005; Schneider *et al.*, 2019). SST1 transports sulfate from the plant cell cytoplasm to the intracellular rhizobia, where sulfate is crucial for protein and cofactor synthesis (Krusell *et al.*, 2005). Moreover, part of the sulfate is incorporated into thiols and is exported back to the plant host (Kalloniati *et al.*, 2015), suggesting that legume–rhizobia mutualism is not limited to N and carbon, but also involves S. S metabolism is the basis of plant growth promotion by several bacterial strains using different mechanisms. While the *Bacillus* sp. B55 emits dimethyl disulfide, which stimulates plant S metabolism and promotes growth (Meldau *et al*., 2013), other bacteria release sulfate from organic sulfate and sulfonates and increase its availability for the plants (Kertesz and Mirleau, 2004). Another *Bacillus* strain, SA187, protects plants against salt stress by induction of sulfate uptake and assimilation (Andrés-Barrao *et al*., 2021), and the S-containing metabolite, camalexin, is necessary for plant growth promotion by a number of bacterial strains (Koprivova *et al*., 2019).

S metabolites, such as GSLs and camalexin, have long been known to be important for plant interactions with pathogenic bacteria as part of plant immunity. However, the interactions of pathogens and S metabolism are broader than that. For example, an *A. thaliana* mutant *pad2* with disrupted GSH synthesis that contains only 20% of the wild-type GSH levels showed reduced accumulation of indolic and aliphatic GSLs and increased susceptibility to fungal and bacterial pathogens (Schlaeppi *et al.*, 2008). Also, when two cultivars of tomato were infected with *P. syringae*, the maintenance of the GSH pool was crucial to resist the bacterial attack (Kuźniak and Skłodowska, 2004). Similarly, the major difference between pumpkin cultivars tolerant or susceptible to fungi was higher GSH levels in the former (Zechmann and Mueller, 2008). In addition, the pathogenic bacterium *Xanthomonas oryzae* pv. *oryzae* produces the sulfated peptide RaxX, which binds to the rice XA21 receptor and activates the immune response (Luu *et al*., 2019). The citrus pathogen *Xanthomonas citri* up-regulates its sulfate transporter upon infection, and the S uptake system affects the virulence of this phytopathogen (Pereira *et al.*, 2017). A different mechanism was discovered through a bacterial transcriptome study. When the pattern-triggered immunity (PTI) was activated in *A. thaliana* plants prior to inoculation of bacterial pathogen *P. syringae*, ‘sulfur metabolism’ was the only bacterial pathway that was significantly up-regulated, compared with bacteria infecting the ‘naive’ host (Lovelace *et al.*, 2018). Among the up-regulated loci were genes for ABC transporters, importing different S sources, such as sulfate, methionine, alkane sulfonates, and taurine, as well as genes for monooxygenases involved in the catabolism of sulfonates and taurine (Lovelace *et al.*, 2018). A similar transcriptomic response of S metabolism genes was observed previously under sulfate starvation in *Pseudomonas aeruginosa* (Kertesz, 2000). Therefore, in analogy with the stimulation of sugar recovery from the apoplast to limit the nutrient sources for pathogens (Yamada *et al*., 2016), it is possible that plants also deplete the apoplast of sulfate, triggering the S starvation response in the bacteria (Wang *et al.*, 2022). Thus, it could be expected that the amount of S in the leaves affects the outcome on pathogen infections. This was not the case for *P. syringae* but was possibly due to high variation within the replicates of the same accession. For the root colonizer *B. glumae* PG1, indeed, a higher bacterial titer was found in accessions with higher S, possibly because sufficient S was available despite the immune response. However, there are many factors that affect the bacterial growth in the plant and most of them also vary in different accessions; therefore, a more focused study using mutants in S uptake and metabolism in the same genetic background would be needed to establish a firm link between S homeostasis and plant–microbe interactions.

### Gene expression and sulfur content

The opposite pattern of expression in the low and high S groups of accessions, combined with sulfate uptake, pin-pointed nine genes involved in GSL and GSH metabolism as being correlated with sulfate content (Fig. 4). Additionally, 11 genes were included in the expression analysis, including previously identified genes that play a quantitative role in foliar sulfate content (Loudet *et al.*, 2007; Koprivova *et al*., 2013), sulfate transporters, because of the observed opposite trend of sulfate uptake in the accessions, and redundant genes to the initial candidates, such as *SOT16* and *SOT17*. Interestingly, the most connected gene in our multivariate network was *SOT18* involved in synthesis of both aliphatic and indolic GSLs. *SOT18* was positively correlated with four genes *SOT17*, *BCAT4*, *GSTF11*, and *PHO2*, foliar S content, foliar sulfate content, sulfate uptake, and indolic GSLs, and only negatively correlated with latitude (Fig. 5). Previous reports of elucidation of metabolite-to-gene networks, specifically in the case of GSL metabolism and desulfoglucosinolate sulfotransferases, showed this to be a solid strategy for the identification of novel gene functions (Hirai *et al.*, 2005).

A negative relationship of phosphate anions and sulfate uptake was observed, so that accessions with higher foliar phosphate have reduced sulfate uptake (Fig. 3C). Therefore, in our expression analysis, three phosphate transporter genes were included (*PHT1;1*, *PHT1;4*, and *PHT1;5*) together with *PHO2*, encoding a ubiquitin-conjugating E2 enzyme that mediates the degradation of PHO1 and PHTs under phosphate starvation (Liu *et al.*, 2012). Our results suggest that GSL and GSH metabolism are tightly linked not only with S metabolism, but also with phosphate transport and signaling (Fig. 5). Previous reports show that during Pi deficiency, phospholipids are replaced by sulfolipids (Essigmann *et al.*, 1998), and vice versa (Sugimoto *et al.*, 2007). Two genes involved in sulfolipid biosynthesis, *SQD1* (*sulfoquinovosyldiacylglycerol 1*) and *SQD2*, are up-regulated under Pi deficiency and regulated by PHR1 (PHOSPHATE STARVATION RESPONSE 1), the main transcription factor regulating Pi starvation response (Bustos *et al.*, 2010). PHR1 also regulates several sulfate transporters, and most prominently *SULTR1;3* expression under Pi deficiency (Rouached *et al.*, 2011). The current finding that phosphate anions levels are negatively correlated with sulfate uptake and foliar S content suggest complex interconnection in the regulation of S and Pi homeostasis dependent on local environmental abiotic and biotic factors.

## Supplementary data

The following supplementary data are available at *JXB* online.

Fig. S1. Natural variation of S content in leaves of *Arabidopsis thaliana* accessions.

Fig. S2. Quantification of anion content in leaves of three groups of *Arabidopsis thaliana* accessions, based on the S content in leaf.

Fig. S3. Sulfate uptake traits in *Arabidopsis thaliana* accessions.

Fig. S4. Linear regression analysis on the relationship of S uptake in opposite groups of *Arabidopsis thaliana* accessions, based on the S content in leaves.

Fig. S5. Clustering analysis was performed on 383 *Arabidopsis thaliana* nutrient-related genes in seven contrasting accessions.

Fig. S6. RT-qPCR analysis in 14 *A. thaliana* contrasting accessions, based on the S content in leaves.

Fig. S7. Quantification of cysteine (Cys) and gluthatione (GSH) in leaves in *A. thaliana* contrasting accessions, based on the S content in leaves.

Fig. S8. Quantification of glucosinolates (GSLs) in leaves of *A. thaliana* contrasting accessions, based on the S content in leaves.

Fig. S9. Linear regression analysis on relationship of S uptake and S metabolites.

Table S1. Ionomics data for 174 overlapping accessions between Baxter *et al.* (2007) and Campos *et al.* (2021) were used to perform PCA, and select three groups of accessions with low, mid, and high S content.

Table S2. RT-qPCR primers used.

Table S3. Loading matrix and eigenvalues of the first 10 principal components (Prin1–10). Ionomics data for 174 overlapping accessions between Baxter *et al.* (2007) and Campos *et al.* (2021) were used to perform PCA.

Table S4. Z-scores of 383 nutrient-related genes in seven accessions with contrasting S content in leaves (expression values transformed from Kawakatsu *et al.*, 2016).

Table S5. *P*-values between low and high S-containing accession groups, for significantly different GSLs. *t*-test (*P*-value <0.05).

Table S6. Correlation matrix of quantified mean traits.

Table S7. *P*-value matrix of quantified mean traits.

Table S8. Pairwise correlations of all quantified mean traits.

erad401_suppl_Supplementary_Tables_S1-S8

erad401_suppl_Supplementary_Figures_S1-S12

## Data Availability

All data are available in the paper and its supplementary data published online.

## Abbreviations:

GSH: glutathione
GSL: glucosinolate
QTL: quantitative trait locus
S: sulfur
