# Supplementary material for: Traits linked to natural variation of sulfur content in Arabidopsis thaliana
Source: J Exp Bot. 2023 Oct 13;75(3):1036–50. doi: 10.1093/jxb/erad401 (PMC10837017; doi:10.1093/jxb/erad401)
Supplement: erad401_suppl_Supplementary_Figures_S1-S12 [file erad401_suppl_supplementary_figures_s1-s12.pdf]

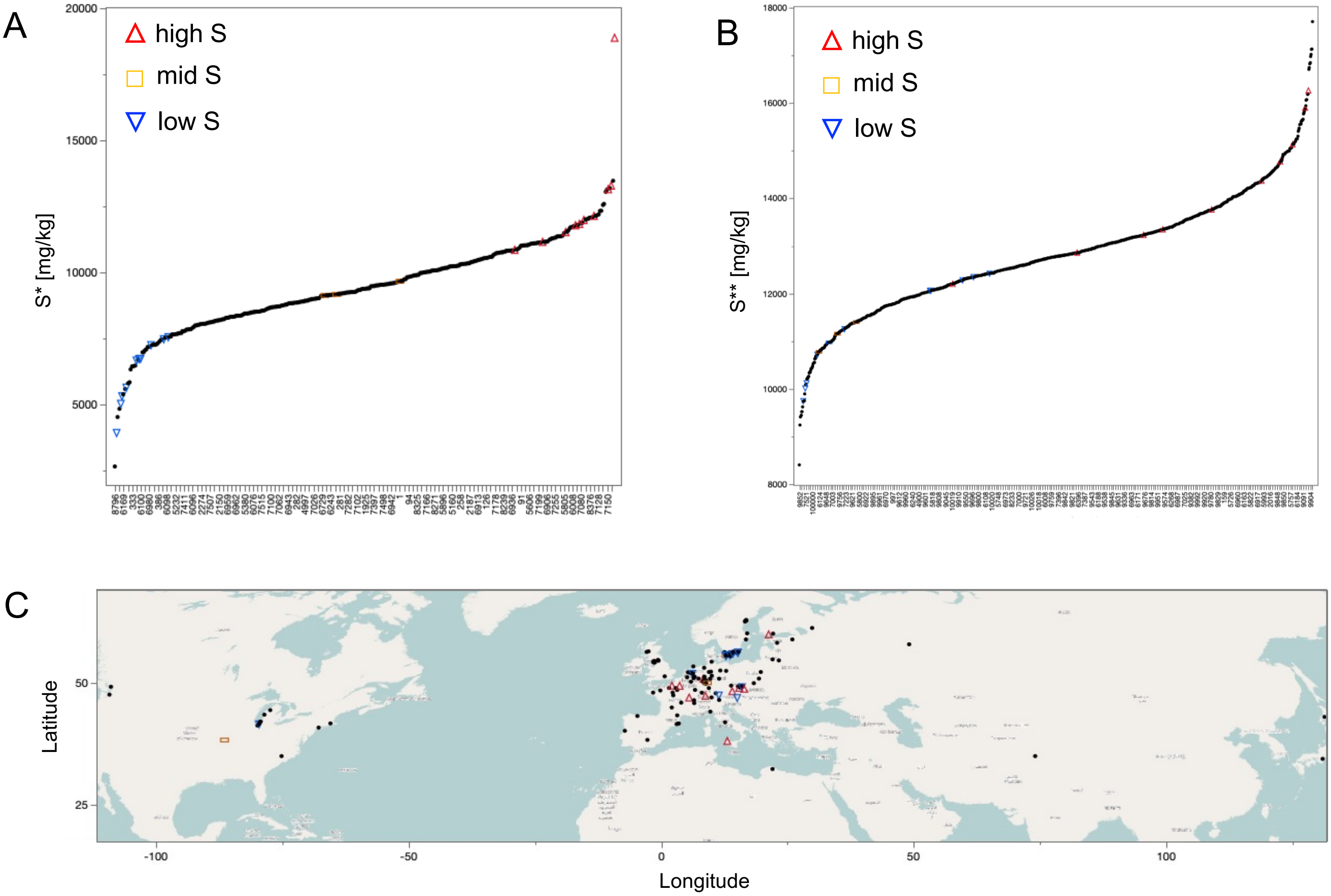

Supplemental Figure S1. Natural variation of sulfur (S) content in leaves in *Arabidopsis thaliana* accessions. A) S content in leaf from Baxter et al., 2007. B) S content in leaf from Campos et al., 2021. C) 174 accessions that overlapped between the two datasets are shown in their natural locations. Accessions that we have chosen based on the PCA (Figure 1), are marked with red up-pointed triangle or accessions with high S content (Com-1, Rak-2, Uod-7, Zu-1, Pa-1, Di-1, Si-0, Tamm-2, Rou-0, Hod), blue down-pointed triangle or accessions with low S content (App1-16, Rhen-1, TDr-18, Jl-3, RRS-7, Gr-5, In-0, Lis-1, Bro1-6, T510, T1110,), and orange square or accessions with mid S content (Col-0 , Kro-0, Kulturen-1).

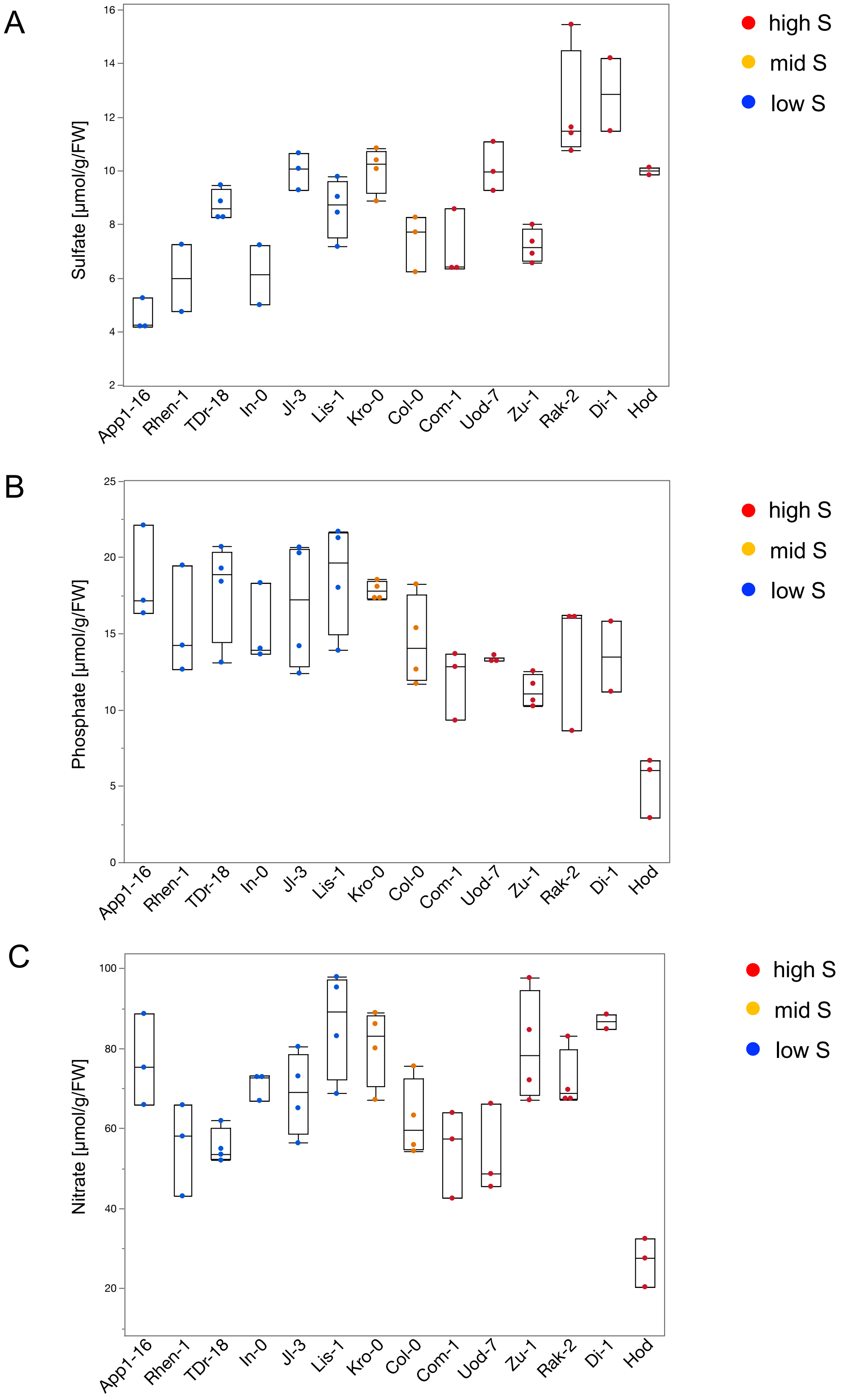

Supplemental Figure S2. Quantification of anions content leaf of 3 groups of *Arabidopsis thaliana* accessions, based on the S content in leaf. A) Quantification of sulfate (SO4) anions. B) Quantification of phosphate (PO4) anions C) Quantification of nitrate (NO3) anions. Plants were grown on vertical plates for 18 days, and shoots and roots were harvested separately. Shoots were used for anions quantification. Ten plants were plated on each plate, and four biological replicates. When harvesting, ten shoots or ten roots from each plate were bulked in one sample; means ±SE are shown, each corresponding to 10 shoots.

A

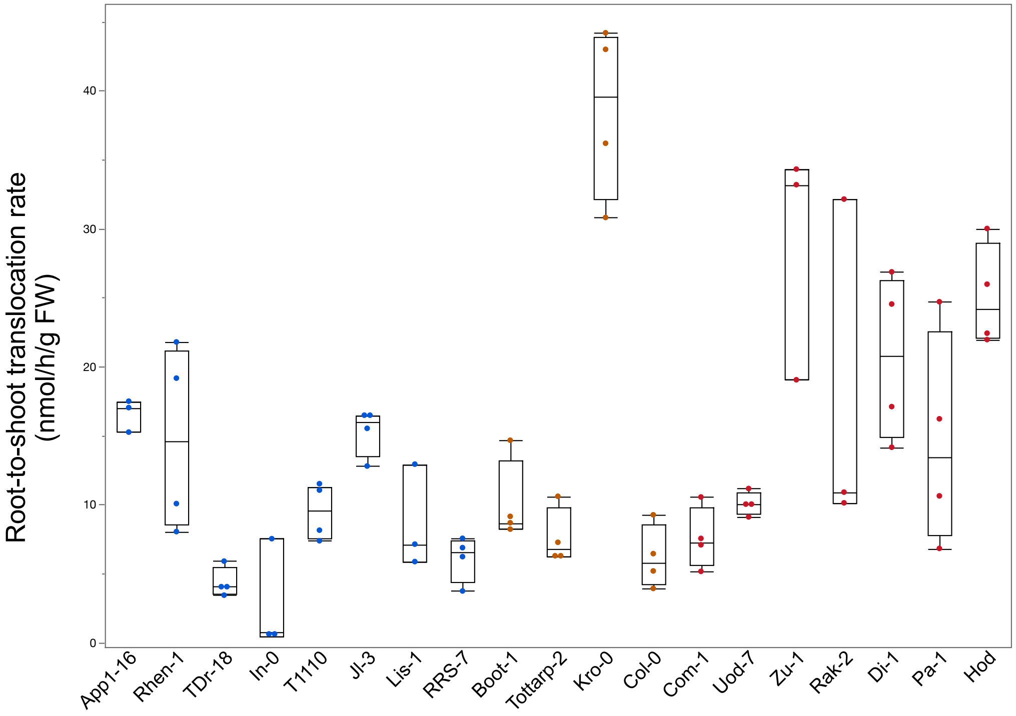

B

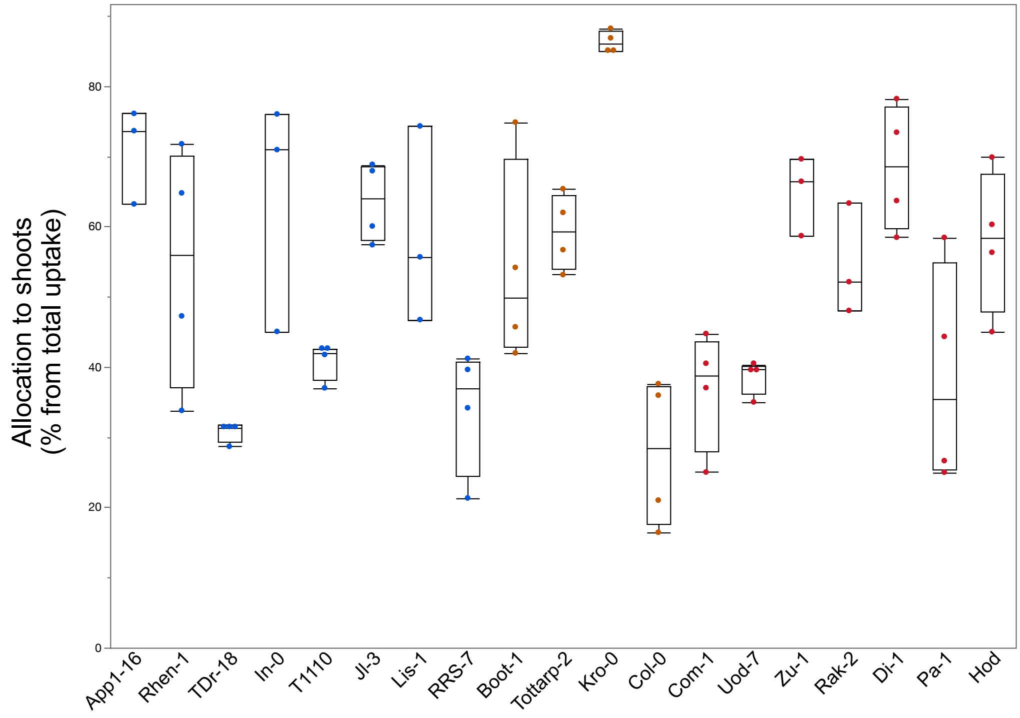

Supplemental Figure S3. Sulfate uptake traits in *Arabidopsis thaliana* accessions. A) Root-to-shoot translocation rate (nmol/h/g FW). B) Allocation to shoot (% of ^{35}S in leaves from ^{35}S taken up). Arabidopsis plants (25 seedlings per replicate) were grown on a nylon net in hydroculture for 14 days, and after incubated with [^{35}S] sulfate for 30 min., and shoots and roots harvested separately and extracted with 0.1 M HCl for radioactivity quantification. Means ±SE (n=3-4) are shown, each corresponding to 25 plants (per replicate).

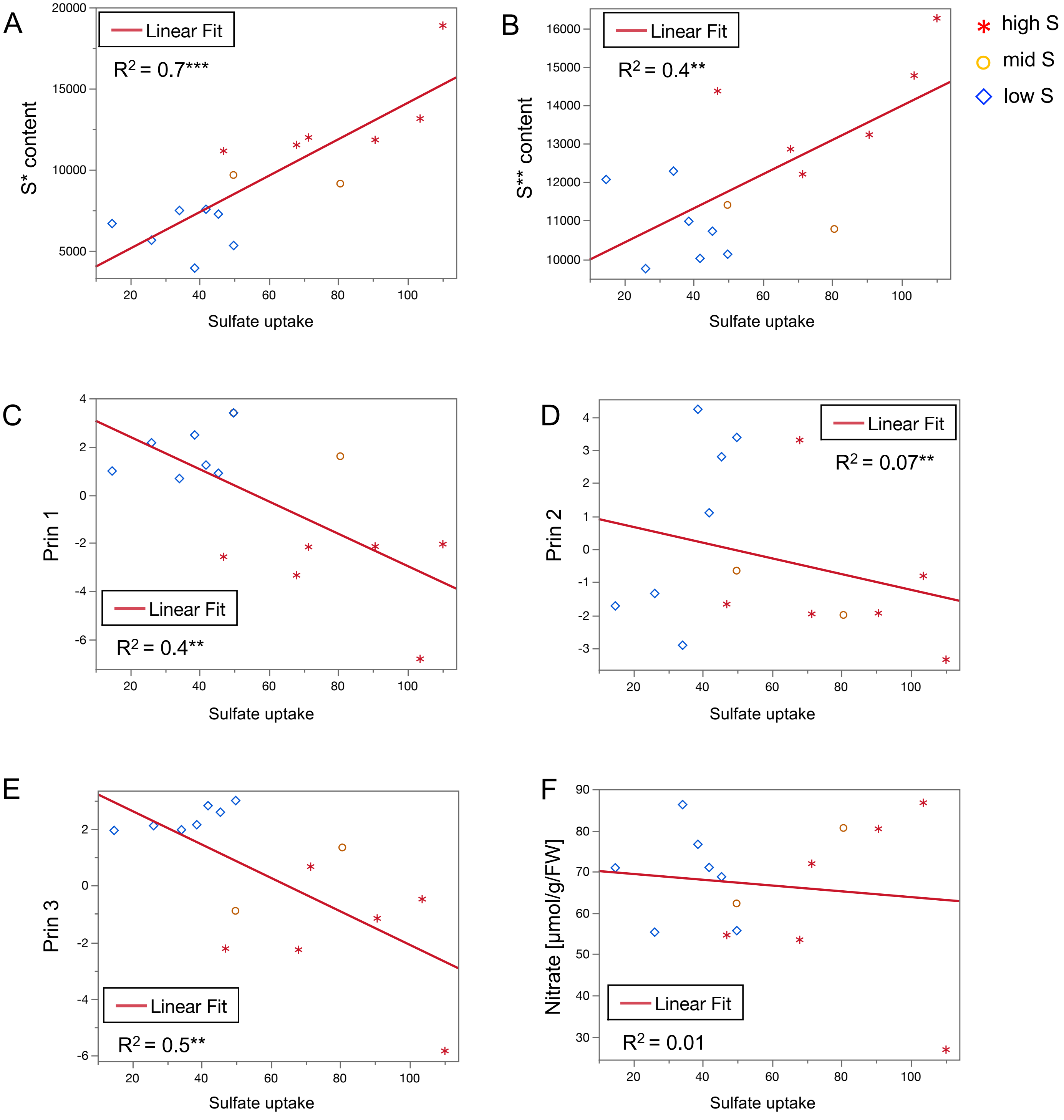

Supplemental Figure S4. Linear regression analysis on relationship on S uptake in opposite groups of *Arabidopsis thaliana* accessions, based on the S content in leaf. A) Linear regression analysis on relationship on S uptake and S content in leaf (from *Baxter et al., 2007). B) Linear regression analysis on relationship on S uptake and S content in leaf (from **Campos et al., 2021). C) Linear regression analysis on relationship on S uptake and Prin1 (principal component 1 from the analysis of overlapping 174 accessions of both dataset). D) Linear regression analysis on relationship on S uptake and Prin2 principal component 2 from the analysis of overlapping 174 accessions of both dataset). E) Linear regression analysis on relationship on S uptake and Prin3 (principal component 3 from the analysis of overlapping 174 accessions of both dataset). F) Linear regression analysis on relationship on S uptake and nitrate anions (NO3). **P<0.05; **P<0.01; ***P<0.001*.

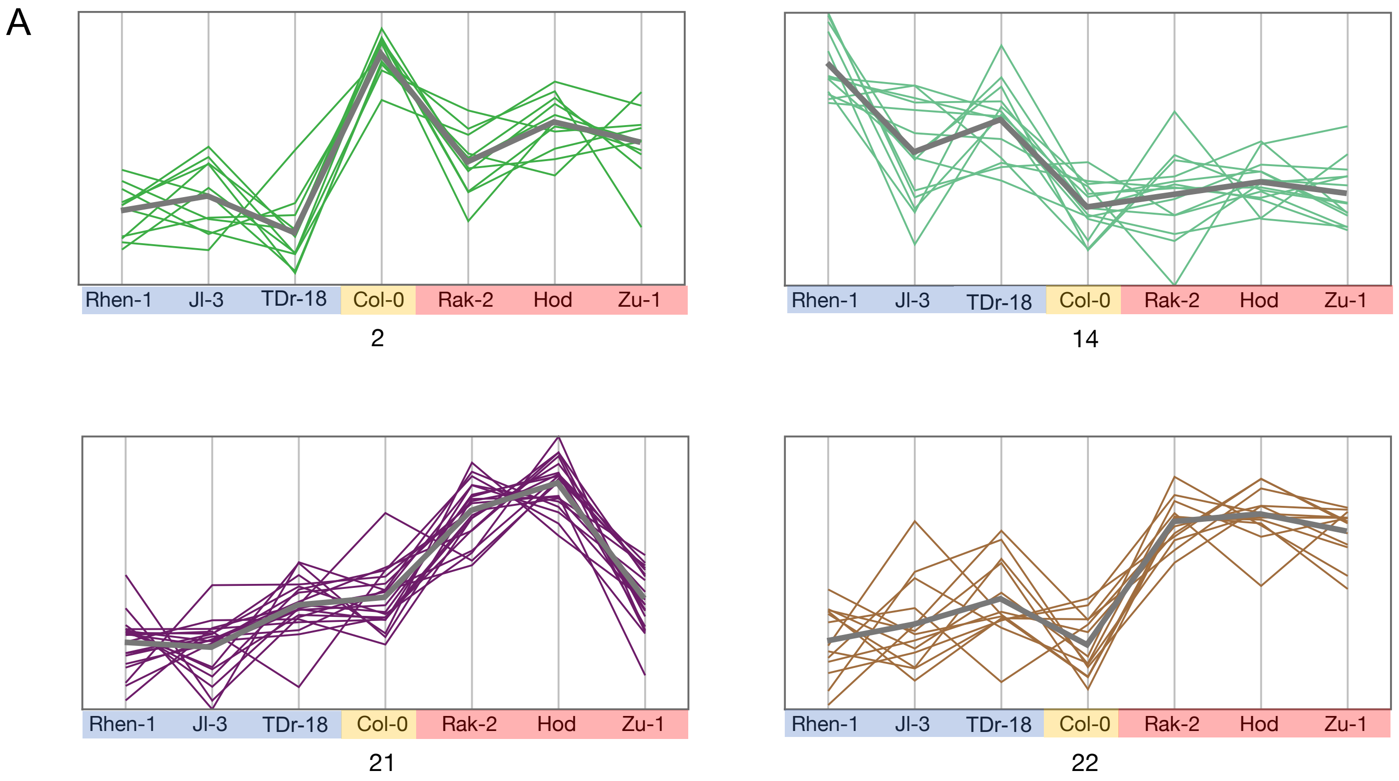

Supplemental Figure S5. **Clustering analysis was performed on 383 *Arabidopsis thaliana* nutrient related genes in seven contrasting accessions.** A) Three clusters showed specific up-regulation of genes in the high S content group of accessions (red color: Rak-2, Hod, Zu-1) e.g. clusters: 2, 21, and 22; and one cluster (14) showed specific up-regulation of genes in the low S content group (blue color: Rhen-1, Jl-3, TDr-18).

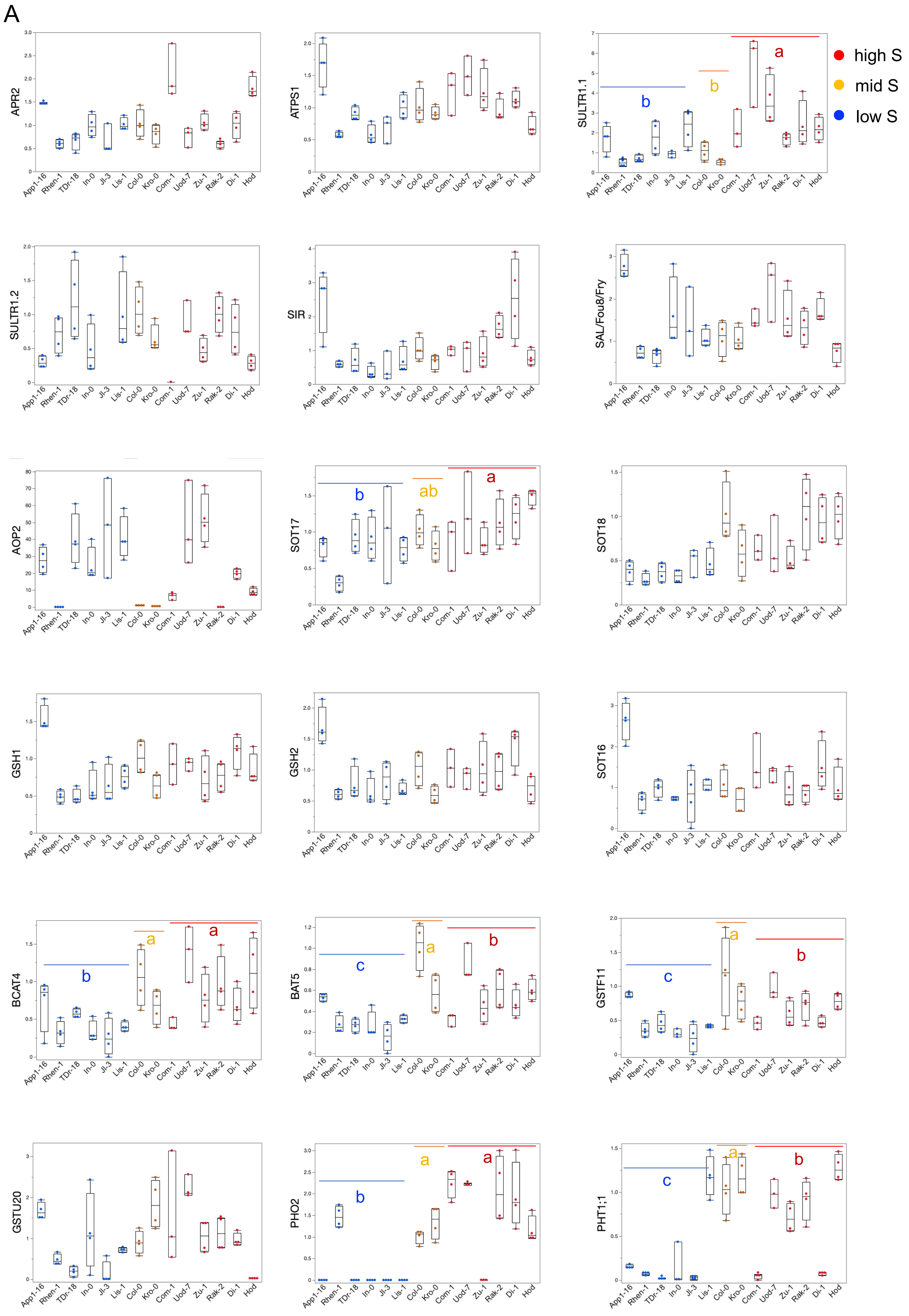
A
high S
mid S
low S
APR2
ATPS1
SULTR1.1
SULTR1.2
SIR
SAL/Fou8/Fry
AOP2
SOT17
SOT18
GSH1
GSH2
SOT16
BCAT4
BAT5
GSTF11
GSTU20
PHO2
PHT1;1
a
b
ab
c
App1-16
Rhen-1
TDr-18
In-0
Jl-3
Lis-1
Col-0
Kro-0
Com-1
Uod-7
Zu-1
Rak-2
Di-1
Hod

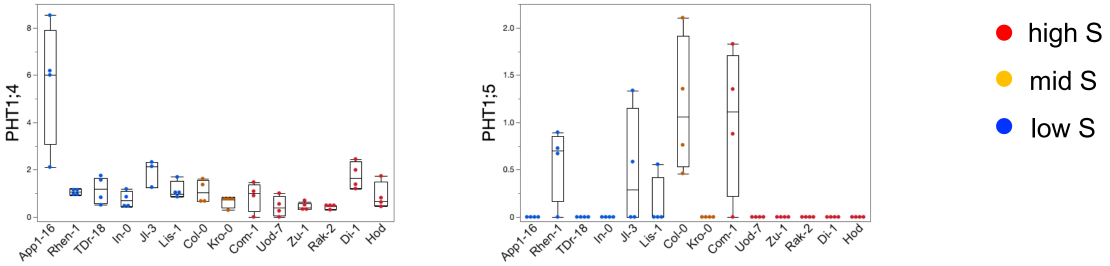

Supplemental Figure S6. **RT-qPCR analysis in 14 *A. thaliana* contrasting accessions, based on the S content in leaf.** A) Expression of 20 genes that have significant R^2 in at least three traits (Fig. 3) were quantified. All transcript levels were quantified by qPCR and normalized to ubiquitin; means ±SE (n=3-4) are shown, each corresponding to 10 shoots; significant differences between the groups are marked with different letter (ANOVA, Tukey test). Plants were grown on vertical plates for 18 days, and shoots and roots were harvested separately. Shoots were used for anions quantification. Ten plants were plated on each plate, and four biological replicates. When harvesting, ten shoots or ten roots from each plate were bulked in one sample.

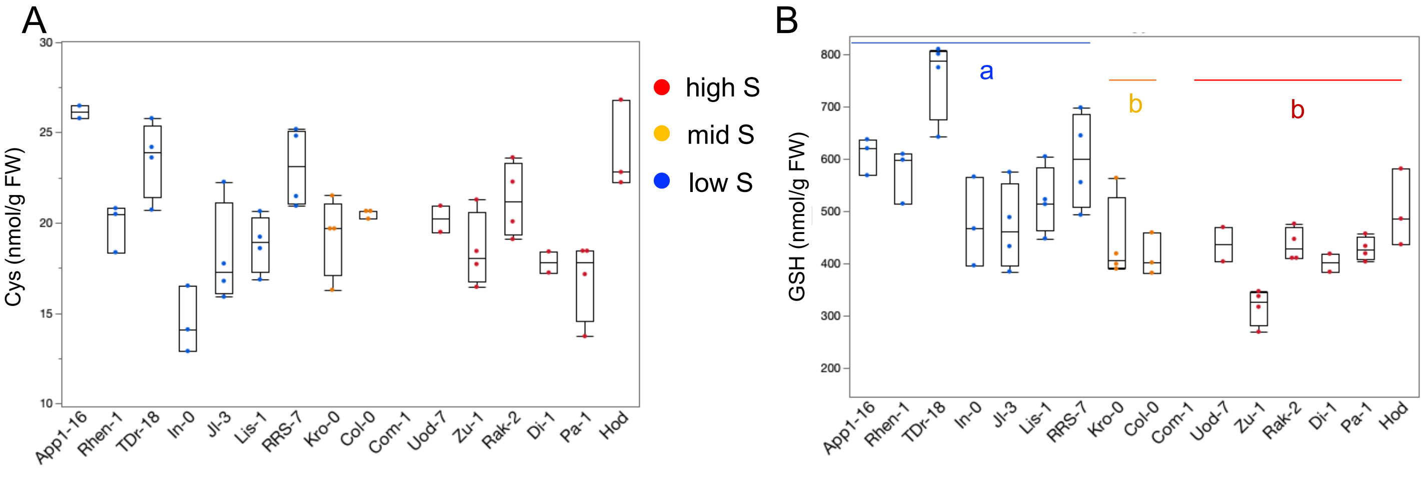

Supplemental Figure S7. **Quantification of cysteine (Cys) and gluthatione (GSH) in leaves in *A. thaliana* contrasting accessions, based on the S content in leaf.** A) Cys content in shoots. B) Total GSH content in shoots. means ±SE (n=4) are shown, each corresponding to 10 shoots. Plants were grown on vertical plates for 18 days, and shoots and roots were harvested separately. Shoots were used for quantification. Ten plants were plated on each plate, and four biological replicates. When harvesting, ten shoots or ten roots from each plate were bulked in one sample. Means shown ±SE (n=3-4) are shown; significant differences between the groups are marked with different letter (ANOVA, Tukey test).

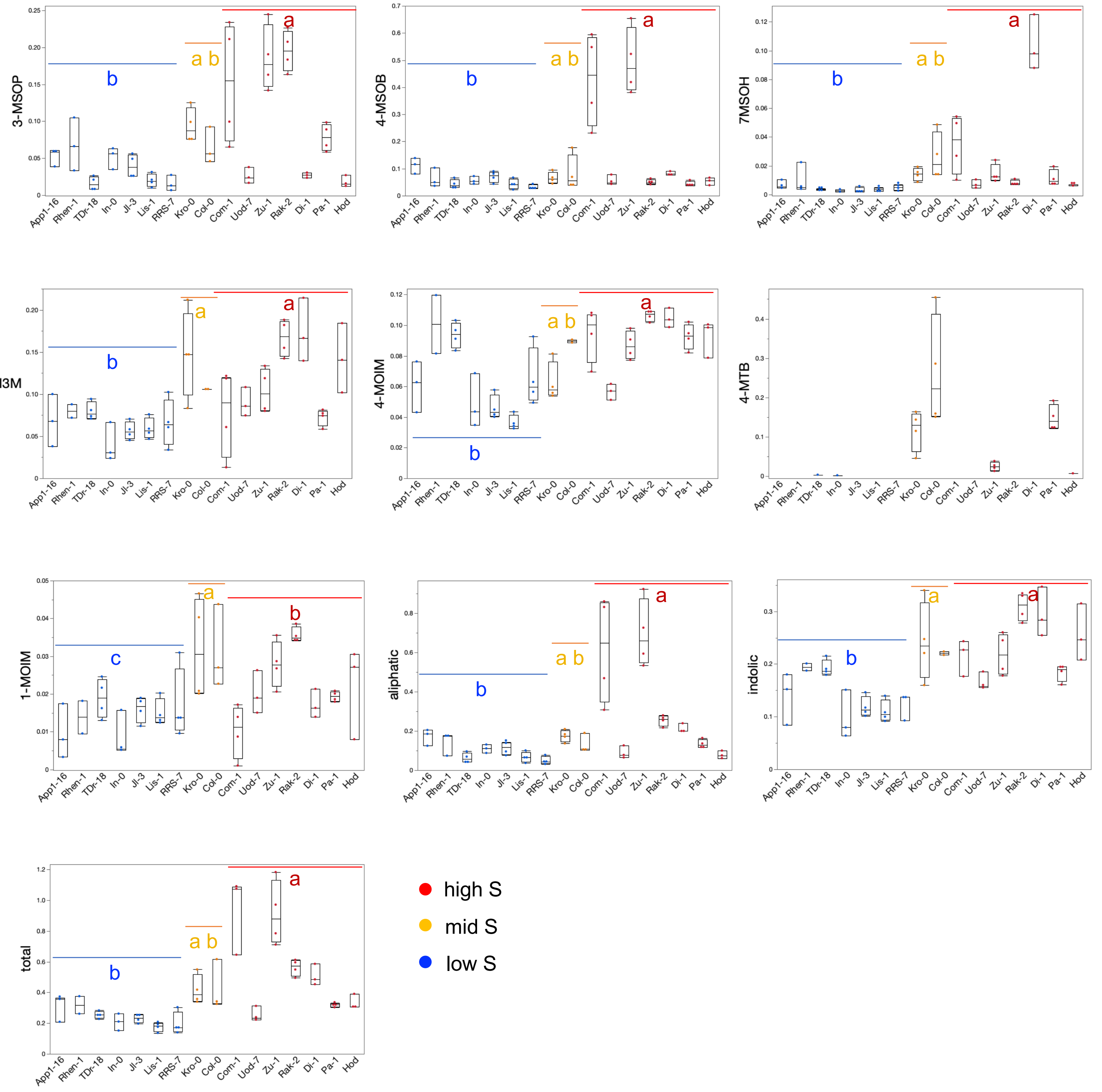

Supplemental Figure S8. **Quantification of glucosinolates (GSL) in leaves of *A. thaliana* contrasting accessions, based on the S content in leaf.** A) GSL content in shoots. Plants were grown on vertical plates for 18 days, and shoots and roots were harvested separately. Shoots were used for quantification. Ten plants were plated on each plate, and four biological replicates. When harvesting, ten shoots or ten roots from each plate were bulked in one sample. Means shown ±SE (n=4) are shown, each corresponding to 10 shoots; significant differences between the groups are marked with different letter (ANOVA, Tukey test). Quantified glucosinolates: 3-MSOP (3-Methylsulfinylpropyl), 4-MSOB (4-methylsulfinylbutyl), 7MSOH (7-methylsulfinylheptyl), 4-MTB (4-methylthiobutyl), I3M (indol-3-ylmethyl), 4-MOIM (4-methoxyindol-3-ylmethyl), 1-MOIM (1-methoxyindol-3-ylmethyl). P-values are listed in Supplemental Table S7.

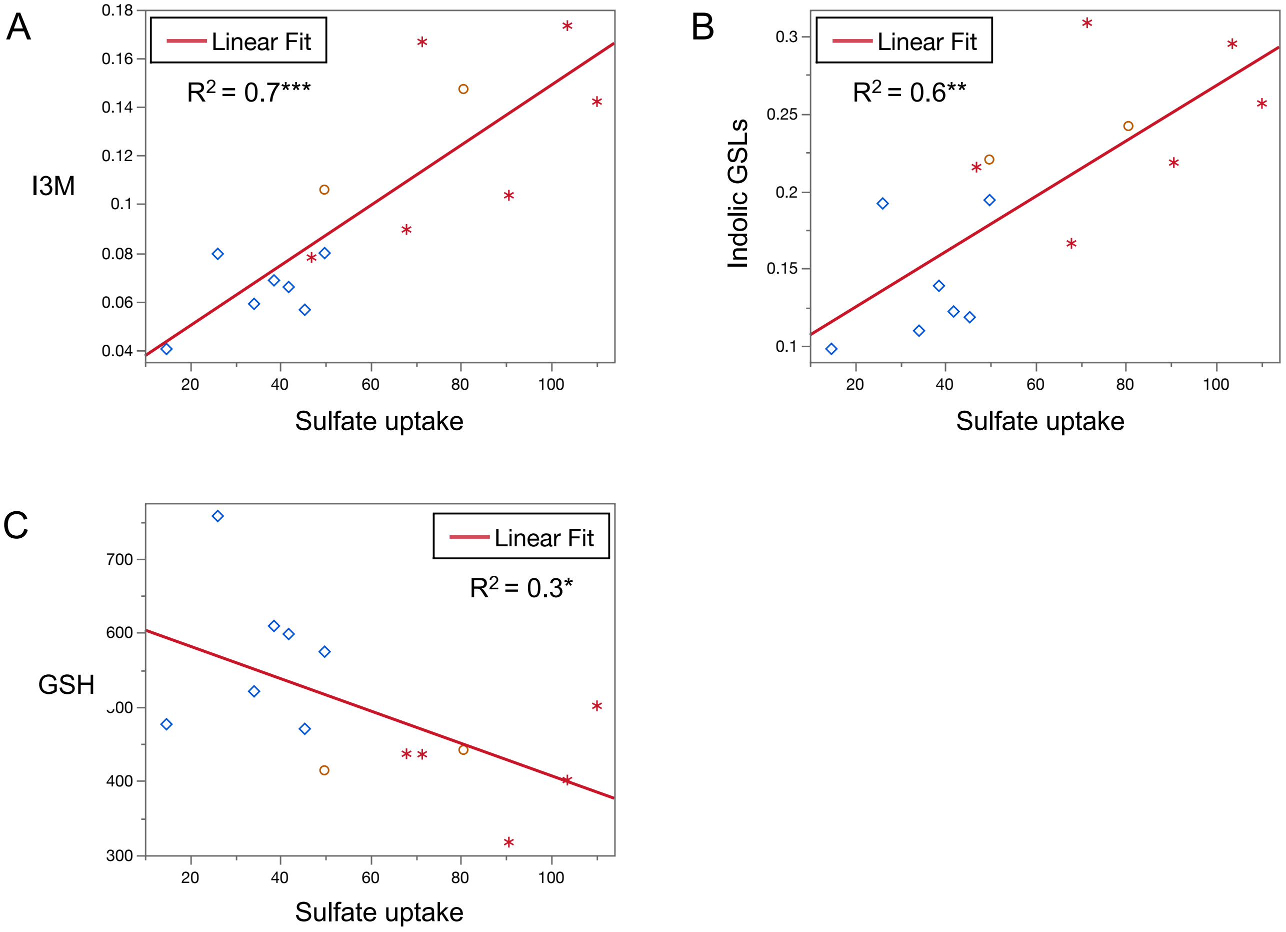

Supplemental Figure S9. **Linear regression analysis on relationship on S uptake and S-metabolites.** A) Linear regression analysis on relationship on S uptake and I3M. B) Linear regression analysis on relationship on S uptake and indolic glucosinolates. C) Linear regression analysis on relationship on S uptake and glutathione (GSH). **P<0.05; **P<0.01; ***P<0.001.*

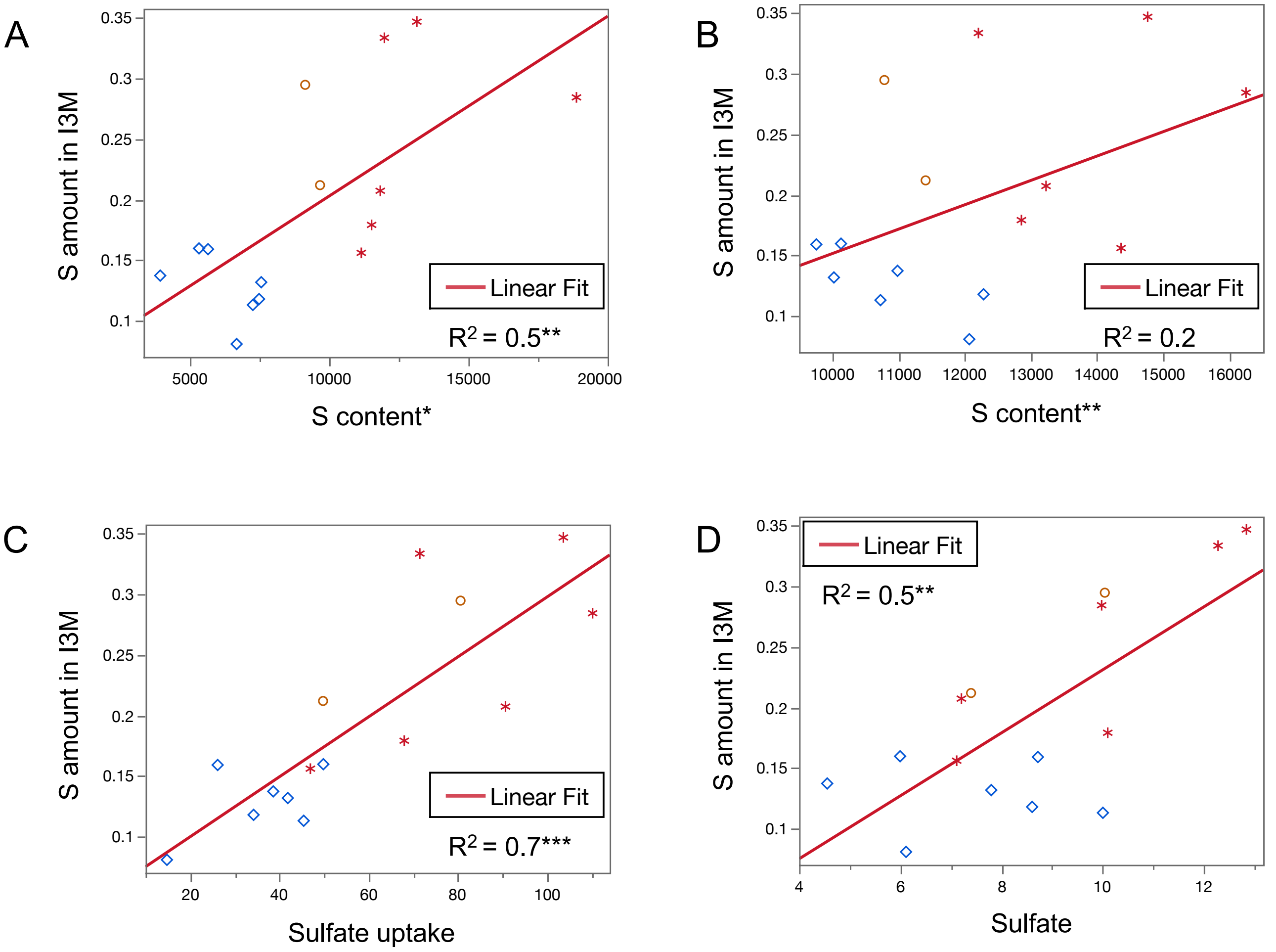

Supplemental Figure S10. **Linear regression analysis on relationship on amount of S atoms in indol-3-ylmethyl (I3M) glucosinolate in opposite groups of *Arabidopsis thaliana* accessions, based on the S content in leaf.** A) Linear regression analysis on relationship on I3M and S content in leaf (from *Baxter et al., 2007). B) Linear regression analysis on relationship on I3M and S content in leaf (from **Campos et al., 2021). C) Linear regression analysis on relationship on I3M and S uptake. D) Linear regression analysis on relationship on I3M and sulfate

$P<0.05$; $^{**}P<0.01$; $^{***}P<0.001$.

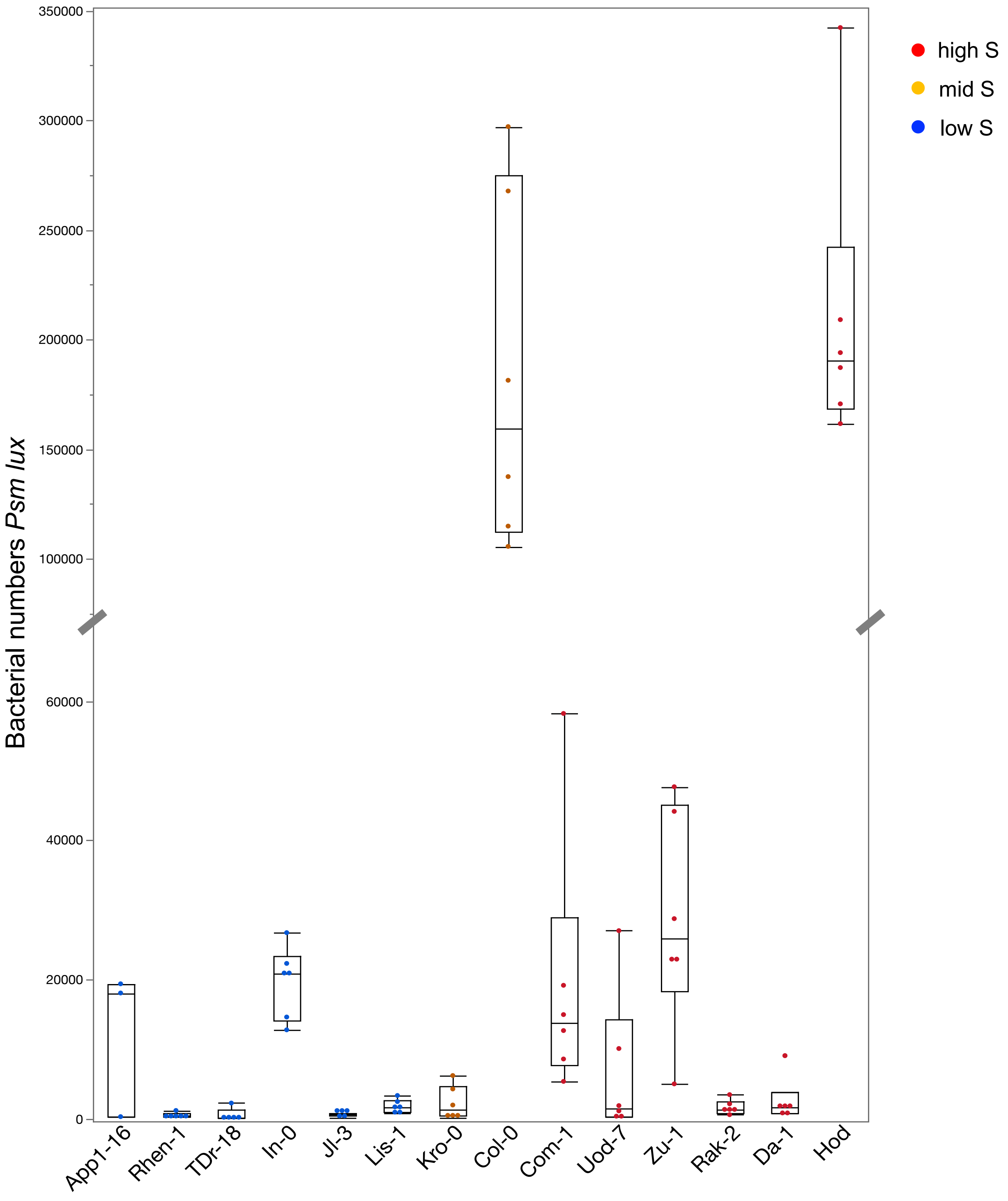

Supplemental Figure 11. **Assessment of plant resistance to *P. syringae.*** 5-week-old plants were inoculated with *Psm lux* (OD600nm = 0.001). Bacterial numbers were assessed at 60 hpi with the bioluminescent *Psm lux* strain and. Data represent the mean ± SE of at least 18 leaf replicates from 6 different plants. Relative luminescence of each ecotype was calculated as a difference of quantified value (infected) and the mean luminescence of all accessions (uninfected).

A

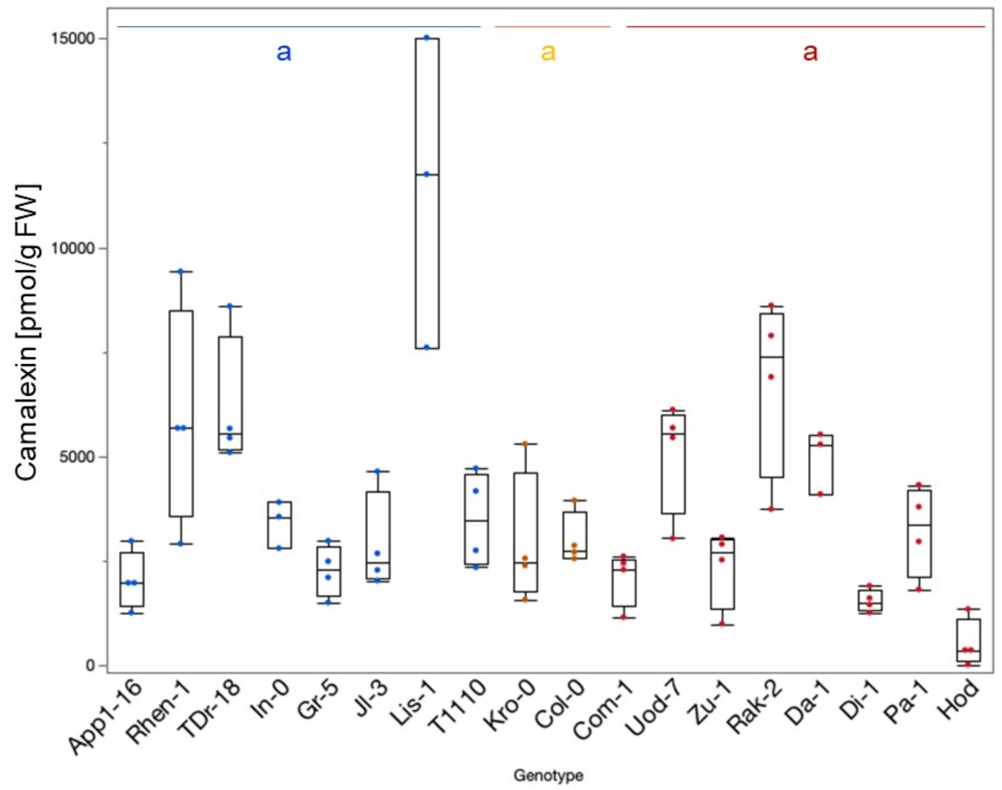

B

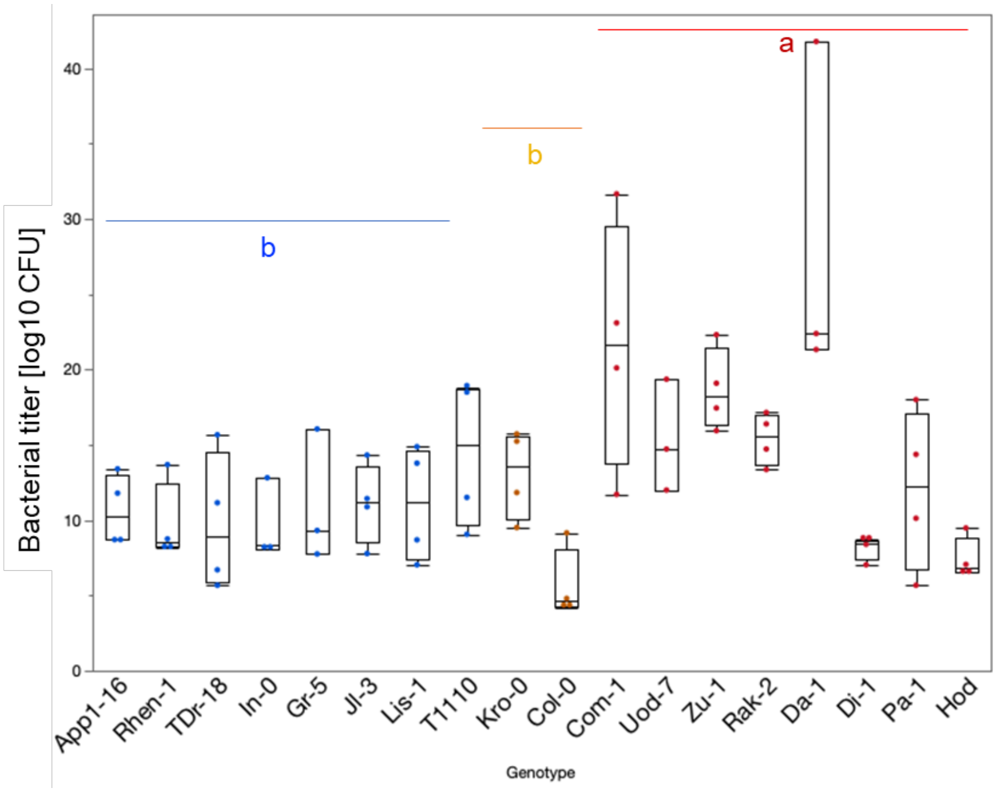

Supplemental Figure 12. Assessment of camalexin synthesis and bacterial titer to *B. glumae* infection. A) Camalexin synthesis quantification. B) Bacterial titer quantification. Arabidopsis plants were grown on a nylon net in hydroculture for 10 days and inoculated in the solution with B. glumae PG1 (BG), or 10 mM MgCl2 as mock, and three days later shoot tissue was harvested for camalexin quantification, and root tissue for bacterial titre quantification. For bacterial titer quantification, DNA was isolated from the roots and subjected to qPCR with primers against *B. glumae* PG1 and the Arabidopsis AT4G26410 gene as a control. Using a previously established calibration between Ct values, OD, and CFU, the qPCR data were expressed as CFU, presented as means ±SD from four biological replicates
